# Beyond-local neural information processing in neuronal networks

**DOI:** 10.1016/j.csbj.2024.10.040

**Published:** 2024-11-13

**Authors:** Johannes Balkenhol, Barbara Händel, Sounak Biswas, Johannes Grohmann, Jóakim v. Kistowski, Juan Prada, Conrado A. Bosman, Hannelore Ehrenreich, Sonja M. Wojcik, Samuel Kounev, Robert Blum, Thomas Dandekar

**Affiliations:** a Department of Bioinformatics, Biocenter, University of Würzburg, 97074 Würzburg, Germany; b Department of Psychology (III), University of Würzburg, 97070 Würzburg, Germany; c Cognitive and Systems Neuroscience Group, Swammerdam Institute for Life Sciences, Center for Neuroscience, University of Amsterdam, 1105 BA Amsterdam, Netherlands; d Experimentelle Medizin, Zentralinstitut für Seelische Gesundheit, 68159 Mannheim, Germany; e Institute of Computer Science, Chair of Software Engineering (Computer Science II), University of Würzburg, 97074 Würzburg, Germany; f Neurosciences, Max-Planck-Institut für Multidisziplinäre Naturwissenschaften, 37075 Göttingen, Germany; g Department of Neurology, University Hospital Würzburg, 97080 Würzburg, Germany; h European Molecular Biology Laboratory (EMBL), 69012 Heidelberg, Germany; i Department of Theoretical Physics I, University of Würzburg, 97074 Würzburg, Germany

**Keywords:** Neuronal field model, Information integration, Neural network, Columnar architecture, Parallel computing, Neuronal oscillations, Visual perception

## Abstract

While there is much knowledge about local neuronal circuitry, considerably less is known about how neuronal input is integrated and combined across neuronal networks to encode higher order brain functions. One challenge lies in the large number of complex neural interactions. Neural networks use oscillating activity for information exchange between distributed nodes. To better understand building principles underlying the observation of synchronized oscillatory activity in a large-scale network, we developed a reductionistic neuronal network model. Fundamental building principles are laterally and temporally interconnected virtual nodes (microcircuits), wherein each node was modeled as a local oscillator. By this building principle, the neuronal network model can integrate information in time and space. The simulation gives rise to a wave interference pattern that spreads over all simulated columns in form of a travelling wave. The model design stabilizes states of efficient information processing across all participating neuronal equivalents. Model-specific oscillatory patterns, generated by complex input stimuli, were similar to electrophysiological high-frequency signals that we could confirm in the primate visual cortex during a visual perception task. Important oscillatory model pre-runners, limitations and strength of our reductionistic model are discussed. Our simple scalable model shows unique integration properties and successfully reproduces a variety of biological phenomena such as harmonics, coherence patterns, frequency-speed relationships, and oscillatory activities. We suggest that our scalable model simulates aspects of a basic building principle underlying oscillatory, large-scale integration of information in small and large brains.

## Graphical Abstract

**Figure:**
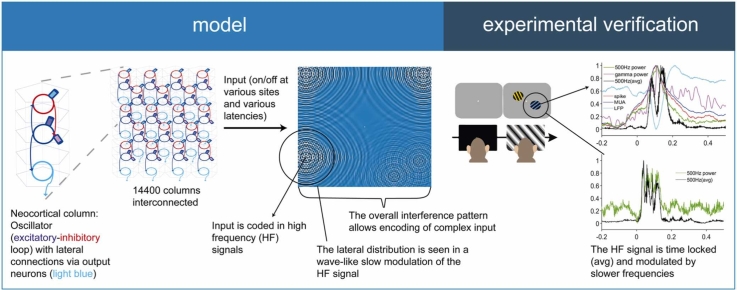

**One sentence summary**

Modelling information integration within the cortex.

## 1 Introduction

In the brain of vertebrates such as mammals or birds, millions of neurons act together in neuronal circuits to enable higher order functions [42]. Connectivity patterns of individual neurons allow the formation of specialized neuronal circuits for specific physiological functions. Due to the complex interplay of up to thousands of active synapses, oscillatory neuronal activity can arise at the single cell level and also appear in neural networks [20], [42]. Such oscillatory activity of the brain, caused by coordinated firing of neurons, contribute to cognitive abilities [16], [20], [62], [66]. For information integration between brain areas, the brain carries a system of neuronal oscillators that can interact at specific frequency patterns [19], [20]. Notably, despite a massive increase in brain size during evolution, the fundamental properties of brain oscillations for information processing within and across neuronal networks are evolutionarily conserved [19], [20], [42].

Over the last decades, the in- and output activity of neurons, neuronal assemblies, and neuronal circuits has been characterized in detail. However, considerably less is known about the emergence of local and global oscillatory activity within large-scale neuronal networks. In order to decode neuronal processing within naturally complex neural circuits, previous work has often been focused on the modeling of specific aspects of neuronal activity [57]. While initially rather simple descriptions of ion fluxes and spiking patterns were described by algorithms [32], [43], other approaches try to learn from virtual networks or even virtual brains how information processing, connectivity, and network structures shape higher-order functions [6], [34], [46], [53], [61], [72]. Another attempt to simulate the neuronal architecture underlying large-scale integration of neuronal activity followed the idea of a holistic integration of information, meaning the representation of the environment as a whole and not as single information streams [66], [67], [11]. This information integration theory (IIT), which is highly controversially discussed [40], claims that a higher order function, such as consciousness, has a physical basis and can be mathematically described [66]. For example, IIT has been applied to resting-state fMRI to quantify consciousness through integrated information across brain networks [50].

Information integration was also modeled using oscillator networks as a representative for the cortex. Such standard oscillator models, starting from a simple oscillator [70], form networks through their interactions. These networks are used to study and suggest various fundamental properties of the brain.

**Previous work which our model takes into account:** Several key studies have significantly advanced our understanding of neural dynamics and information processing in the brain. Oscillator networks have significantly advanced our understanding of neural dynamics and information processing, providing a unified framework that links macroscopic and microscopic brain activity across perception, behavior, and imaging data [17]. Simple models reproduce how geometric constraints and structural connectivity shape brain function, emphasizing the role of anatomical architecture and connectivity in influencing neural oscillations [55] and showing that cortical resonance frequencies could correlate with network size and connectivity [38]. Moreover, coupled oscillator networks are studied in both neuroscience and machine learning [25], [36], [58], [33]. Effenberger et al. [25] explore in their preprint fundamental computational principles of oscillations in recurrent neural networks and outline the role of amplitude, phase, and frequency in information integration and learning. Further, Hughes et al. [33] propose the use of wave physics as an analog recurrent neural network, demonstrating how wave systems efficiently process time-varying signals for tasks like classification.

In addition, other models have been continuously developed, leading to recent innovations such as graph neural fields [8], which consist of systems of stochastic integro-differential equations on graphs. Continuous neural fields fit neuronal observations, allowing for the estimation of the graph neural field model on a high-resolution connectome graph [7]. Oscillator models are also studied to investigate synaptic plasticity when cortical populations interact [39]. Recurrent oscillator networks have enhanced pattern recognition [27] and improved time-resolved sorting of stimuli [24].

In the animal kingdom, oscillations are observed at various scales, from large cortical waves to localized neural circuits, and even simple neural architectures can produce oscillatory behavior. Here, we introduce a simplified neuronal field model that offers significant flexibility while maintaining simplicity. This model allows for the emergence of broad-frequency bandwidth processing, wave interference patterns, and complex information integration, as well as encoding and decoding of stimuli. External stimuli are stored in our model by a holography-like process of interfering waves. This means that a ground oscillation is present on which the modulatory input (the different external stimuli) interferes. The resulting wave pattern is then accessible to all participating columns of the model and contains and integrates the full information of different external stimuli. Its simplicity enables a deeper understanding of the principles underlying cortical processing.

**Specific achievements of our model:** Stimulated and building on own (since 2019) and noting other prior efforts (Lea-Carnall et al. [38]*,* Balkenhol et al. [13]*,* Breakspear [17]*,* Hughes et al. [33]*,* Pang et al. [55]*,* Effenberger et al. [25], Nemirovsky et al. [50])*,* our approach unifies several aspects of brain dynamics, offering key innovations such as mathematical flexibility through adjustable coupling constants, precise control over neural dynamics and connectivity; functional application in encoding and decoding real-world data, like images and sounds through neural oscillations, making it practical for simulations; and the bridging of hierarchical dynamics, linking local interactions with global oscillatory behavior across scales. Moreover, the model integrates with neurophysiological data, simulating oscillations observed in brain activity, making it a valuable tool for exploring cognitive tasks. Our model can even reproduce signaling patterns of real stimulus presentation tasks, such as stimulus-induced and evoked potentials in the visual cortex, which are known to represent active stimulus processing and higher-order brain processes associated with perception. By combining multiple aspects of previous models, this comprehensive environment offers both theoretical insights and practical applications in neural simulations.

Our model offers a novel combination of:

(i) a deliberately reductionistic, simplistic model of neuronal oscillators
(ii) highly integrative properties for sensory stimuli
(iii) surprisingly good agreement with monitored physiological states of the brain

**Fig. 1:**
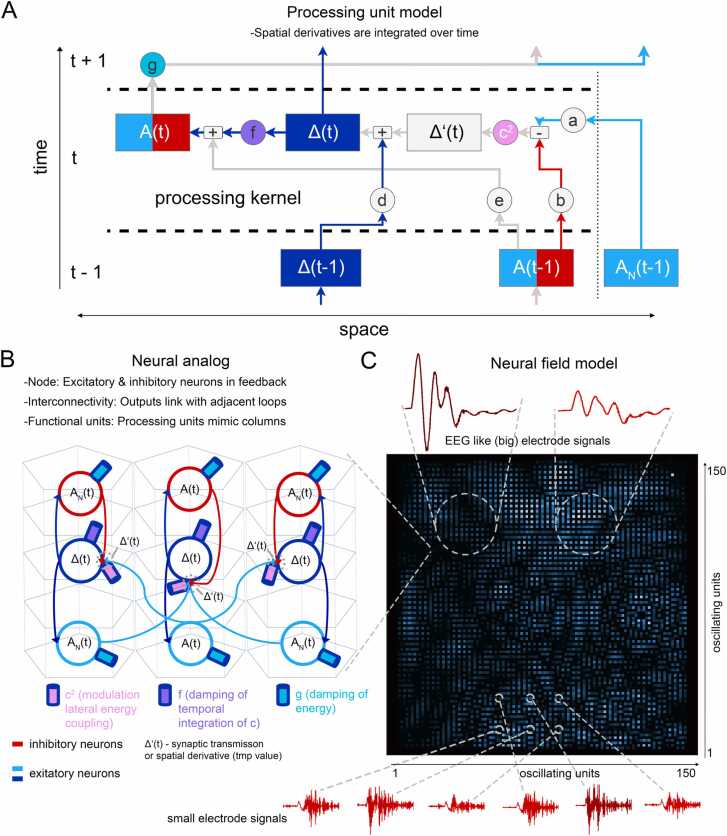
**Model outline**. **(A)** The model relies on the unification of activation and inhibition within a virtual oscialltor (node) and the interconnections with neighboring nodes. Three processing steps are central for processing and modulating the model: Δ’(t) (amplitude deviation of the own amplitude à impulse), Δ(t) (integration of impulse à speed) and A(t) (integration of the speed à amplitude or distance). The three processing steps can be modulated by several parameters (a-g). **(B)** The model represents virtual oscillators and interconnects them laterally. **(C)** Information is distributed over the entire model and is available at any node over time. Each pixel represents one node (as shown in A and B). The amplitude changes over time can be recorded by different electrode sizes, small electrodes (one node), and big electrodes (many nodes), thus representing the sum of many small electrode recordings (EEG-like signals).

To test our hypothesis, whether model simplicity can model biological complexity, we built up a reductionistic neuronal network model (Fig. 1). In the model, a node (a microcircuit) with an inhibitory feedback architecture represent the basic processing unit (Fig. 1A). By implementing lateral connections between neighboring model nodes representing biological microcircuits of the cortex (lateral connections typical in layer II-IV of six in the neocortex, [59], [49]; Fig. 1B), we simulated the connectivity between the nodes of the neuronal network. Exploiting the advances in power for parallel computing allowed us to apply simple rules to a scalable, large simulation of network dynamics. By this building principle, the same frequency-coded information, modulated in a wave-like fashion through the lateral connections, always reaches all participating columns and creates a holistic representation of the processed information (Fig. 1C). We show that a high number of interconnected nodes (microcircuits) is essential for stable processing of a high information content. Importantly, we can demonstrate that the model creates oscillatory activity patterns like those observed in in vivo recordings of the visual cortex of humans and monkeys in a visual perception paradigm.

## 2 Materials and methods

### 2.1 Mathematical description of the information processing model

The main algorithm processes information encoded into independent “activation” amplitudes at each node of a square grid, evolving them discretely in time. The key ingredient of the model involves the evolution of the activation $A\left(t\right)$ at a node based on “lateral feedback” from its neighbors in terms of the average difference between the activation of the node and those of its neighbors, a quantity also known as the grid Laplacian. At a time step *t*, the activation $A\left(t\right)$ increases by the lateral feedback variable $\Delta \left(t\right)$ that incorporates the feedback from neighboring nodes not only in terms of the average activation difference at time t, but the sum of all activation differences in all previous time steps— in other words, the time integrated grid Laplacian. The time-integration of activation-differences in the lateral feedback causes “temporal feedback” such that the activation gradients at a given time affect the time-evolution of activations at later times.

The model can now be described in terms of the evolution of the activation $A\left(t-1\right)$ at a given node to its activation $A\left(t\right)$ at the next time step, in terms of the activations of neighboring nodes denoted by $A_{\mathrm{Ni}}\left(t\right)$ (where Ni=N1,N2⋯Nk denote the k neighbors of the node in question): First we calculate the average activation difference, or grid Laplacian, at the node given by $\Delta^{\prime }\left(t\right)=\frac{1}{k}\sum_{\mathrm{Ni}}^{\mathrm{Nk}}\left(A_{\mathrm{Ni}}\left(t-1\right)-A\left(t-1\right)\right)$. The grid Laplacian at time t is now added to the time-integrated lateral feedback $\Delta \left(t\right)$ such that it incorporates the feedback from activations of neighboring nodes at different times. The evolution can be succinctly described by the steps:

#### 2.1.1 Main algorithm

1. $\Delta^{\prime }\left(t\right)=\frac{1}{k}\sum_{\mathrm{Ni}}^{\mathrm{Nk}}\left(A_{\mathrm{Ni}}\left(t-1\right)-A\left(t-1\right)\right)$
2. $\Delta \left(t\right)=\Delta \left(t-1\right)+\Delta^{\prime }\left(t\right)$
3. $A\left(t\right)=A\left(t-1\right)+\Delta \left(t\right)$

Modulation outside of the main loop is done on $E_{N}\left(t-1\right).$ It is important to distinguish between those variables, as the processing units are queried several times within a processing step. The main algorithm results in time evolution of the Energy levels given by Eq. 1**:**

(1) $$A\left(t\right)=A\left(t-1\right)+\Delta \left(t-1\right)+\frac{1}{k}\sum_{\mathrm{Ni}=N1}^{\mathrm{Nk}}\left(A_{\mathrm{Ni}}\left(t-1\right)-A\left(t-1\right)\right)$$

### 2.2 Modulation of our information processing model

It turns out a slight generalization of Eq.1 makes it possible to simulate a wider range of processing states (e.g. waking and diseased states). First, we use a modulation constant $c^{2}$, the lateral feedback coupling, such that the activation increases by $c^{2}\Delta \left(t\right)$ instead of $\Delta \left(t\right)$ at each time step. Finally, we add damping constants f and g. At each step, the lateral feedback $\Delta \left(t\right)$ is suppressed by a factor of $\left(1+f\right)$ controlling the fact that the evolution depends more strongly on the grid Laplacian at recent times, compared to times longer back in the past. Finally, the activations $A\left(t\right)$ are themselves suppressed by a damping factor of $\left(1+g\right)$ at each time step — so that the activations can settle down to a resting state in absence of an external stimulus. Note that setting $c^{2}=1$ and the damping constants f=g=0 takes us back to the main algorithm without modulations.

#### 2.2.1 Main algorithm with modulation

The following steps show the generalization of Steps 1–3 of Eq. 1 in previous section to the case with the modulations described above, as shown in Fig. 1A, C.

1. $\Delta^{\prime }\left(t\right)=\frac{1}{k}\sum_{\mathrm{Ni}}^{\mathrm{Nk}}\left(A_{\mathrm{Ni}}\left(t-1\right)-A\left(t-1\right)\right)$.

2. $\Delta \left(t\right)=\Delta \left(t\right)+c^{2}\Delta^{\prime }\left(t\right)$.

3. $\Delta \left(t\right)=\frac{\Delta \left(t\right)}{1+f}$.

4. $A\left(t\right)=A\left(t-1\right)+\Delta \left(t\right)$.

5. $A\left(t\right)=\frac{A\left(t\right)}{1+g}$.

The resulting modulated main algorithm steps 1–5 above-) results in the time-evolution of Energy activations at each node given by

(2) $$A\left(t\right)=\frac{A\left(t\right)}{1+g}\left(A\left(t-1\right)+\frac{1}{1+f}\left(\Delta \left(t-1\right)+\frac{c^{2}}{k}\sum_{Ni=N1}^{Nk}\left(A_{\mathrm{Ni}}\left(t-1\right)-A\left(t-1\right)\right)\right)\right)$$

### 2.3 Parallel computing of the beyond local information processing model

In this simulation, we employed a basic quadratic grid topology as a starting point due to its simplicity and ease of implementation. Although manifold topologies are feasible, our model initially utilized a nearest-neighbor grid structure to demonstrate fundamental principles. Nevertheless, the model was designed to accommodate adjustments that would facilitate the investigation of both short-range and potential long-range synaptic connections, along with the incorporation of preferred directionalities within the neural network. It should be noted, however, that the findings presented in this manuscript are derived from simulations based on the grid architecture. This model was scaled up to include 400,000 nodes distributed across four distinct CPUs. Each measurement was repeated five times per CPU. The different types are a 2-core Skylake laptop (Intel i7–6600U), a 10-core Haswell (E5–2650V3), a 12-core Broadwell (E5–2650V4), and a 4-core Skylake architecture (Intel E3–1230V5) system. In total, 970 measurements (194 grid sizes × 5 measurement repetitions) were conducted for each system. The simulation is written in C, while the benchmarking and analysis script is written in Python. The source code for both is open-source and available on GitHub: https://github.com/DescartesResearch/BrainSimulation.

We utilized NetLogo 6.1.1, a multi-agent programming environment, to construct and simulate the neural network [69]. NetLogo was also used for visualization of network dynamics and user-friendly interaction with the simulation.

### 2.4 Signal analysis

Analyses throughout the manuscript were computed with Matlab R2021a (MathWorks, http://www.mathworks.de/products/matlab/). The simulation and the Matlab analysis scripts are provided in the supplementary material.

#### 2.4.1 Correlation network analysis and resonance analysis

We performed correlation network analysis as described earlier [28]. Each location of a node (i) was correlated with every other location in the model (j). The correlation coefficient r between all locations was calculated as follows:

(3) $$r\left(i,j\right)=\frac{\left(\left\langle x_{i}\left(t\right)x_{j}\left(t\right)\right\rangle -\left\langle x_{i}\left(t\right)\right\rangle \left\langle x_{j}\left(t\right)\right\rangle \right)}{\sigma \left[x_{i}\left(t\right)\right]\sigma \left[x_{j}\left(t\right)\right]}$$

where $\sigma^{2}\left[x_{i}\left(t\right)\right]=\left\langle x_{i}^{2}\left(t\right)\right\rangle -{\left\langle x_{i}\left(t\right)\right\rangle }^{2}$ and $x_{i}(t)$ is the signal of a column at location i. The analyzed signals represent 3000 ms (data points) and < .> represents the average over this time period. The Matlab function corrcoef() was used to calculate r(i,j). The correlation networks determine direct interaction partners based on the cutoff, ρ, of the correlation coefficient. Similar to r the cutoff can be positive ρ + or negative ρ-, thereby differing between two types of networks: positive correlated and negative correlated networks.

In this study, we only analyzed the positive correlation network with a ρ + of 0.07. Based on ρ + we estimated the degree k, the number of direct neighbors, for each column. The network can then be represented as degree distribution P(k), as shown in Fig. S5 for different model sizes.

The script *neoNoLIP Ising like dynamics.nlogo* was used to simulate network activity. The scripts *correlation_network_analysis_sub.*m and *analyse_criticality_sub.m* were used for the correlation analysis.

#### 2.4.2 Frequency space analysis

Our methodological approach allows analysis of temporal and spatial wave patterns through the exploration of frequency spaces. The computational analysis of frequency domains was carried out using a Fast Fourier Transform (FFT) algorithm, implemented via the *fft()* function in Matlab. The custom scripts for Fourier analysis are: - *fft_peak_analysis_sub.m*, *peakstatsMEA.m*, and *peakstatsEEG.m* for peak identification.

To identify temporal dynamics in the frequency space, a short-time Fourier Transform (STFT) divides signals into sections of distinct lengths and overlaps. The resulting time-frequency plots indicate changes in the frequency compositions, e.g., after stimulus onset, in baseline activity. The STFT of the signals was executed with the Matlab function *stft()*. The full analysis script is *sfFT_sub.m*.

#### 2.4.3 Wave speed analysis

We performed a wave speed analysis. The input location was set to x = 0 and y = 0. The time was measured of how long a wave traveled from the input location to a certain site (x = 100 and y = 0) of the model. The maximum frequency that could be generated by the model was determined using the script *fft_peak_analysis_sub.m*. The measurement was repeated for different values of *lateral* amplitude *coupling*. The scripts for full analysis are *wavespeed_analysis_NI_vs_frequency.m* and *wavespeed_analysis_hz_vs_speed.* In order to analyze the speed of waves, the input parameter can be adapted. For this study, we calculated the speed of wave propagation in response to a periodic peak frequency of 7 Hz.

#### 2.4.4 Coherence analysis

The coherence analysis can be used for analyzing the spatial propagation of frequency and phase-encoded information. We used this for testing coherence between activity at different model distances. We computed the coherence analysis with the Matlab function *mscohere()*. The signals of model locations with different distances were (in mm): 0.5, 2, 4, 8, 16, 32. The full coherence analysis script is *coherence_sub.m*.

#### 2.4.5 Lempel Ziv Complexity

In this study, we explore the Lempel Ziv Complexity (LZC) as a potential metric for various states of the neuronal network. We applied LZC to our field model to measure signal complexity. The signals were converted into binary sequences by comparing each data point against the median signal value. We then employed the *lempelzivEnc()* function in MATLAB to assess the binary sequences. The MATLAB script *LZC_analysis_EEG.m* contains the complete analysis process.

### 2.5 *In silico* grating stimulation of the columnar beyond local model

The grating simulation incorporated a 3 s time period presented 100 times (corresponding to 100 trials). The grating was defined as continuous input over a stimulation period of 1 s. Before grating onset and after grating offset a 1 s period without input was added. The grating had a radius of 10 mm and had four stripes with a constant input of 10 mV and four stripes with a constant input of −9 mV (compare fig. S8A and **video 11**). To test the basic response properties of our model, we chose a simple non-moving grating. The simulations were computed using virtual EEG-electrodes with varying distance to the stimulus. The simulated EEG-electrode, with a radius of 2.5 mm, was either at the same location as the grating stimulus (diameter of 5 mm), 12.5 mm away, or 40 mm away. The size of the model was 75 × 75 mm (150 ×150 columns) and the amplitude coupling parameters were set to *modulation of lateral amplitude coupling (c)* of 2.6655, *damping of the amplitude (g)* of 0.0001, and *damping of the temporal integration of amplitude coupling (f)* of 0.01. The full length of a grating experiment was 300 s, including 100 grating onsets (trials).

The analysis followed the analysis of the in vivo data as closely as possible, which is described in detail below. There was one exception: since there was no delay between stimulus onset and model response, we chose the time window for baseline correction to be between −0.25 to −0.1 s, the prestimulus period as −0.6 to −0.1, and the poststimulus period as −0.1 to 0.4.

### 2.6 *In vivo* grating experiment: V1 microelectrode recording in a macaque monkey

Statistical analysis is shown over trials. Note that we only analyzed available animal data which have been published previously (see data availability statement; all procedures were approved by the ethics committee of the Radboud University, Nijmegen, NL).

Behavioral paradigm and visual stimulation. A trained male macaque monkey participated in a study using a change detection paradigm. Trials started when the monkey touched a bar to start the experiment. A fixation point (∼0.2° diameter) lit up and gaze had to be held within a small window around the fixation point, as previously described [71]. Consecutively, a prestimulus baseline (1 s) started, followed by the appearance of two stimuli placed in different visual quadrants (fig. S8B). We refer to this time point as stimulus onset. The stimuli were luminance gratings with a contrast of 100 %, a diameter of 2–3°, a spatial frequency of 1–2 cycles/°, and a temporal frequency of 1–2°/s. The grating within the receptive field (RF) of the recorded neurons always had the preferred orientation and was moving. The grating outside the RF, on the opposite side of the fixation point, was either presented in the preferred or anti-preferred orientation and was also moving. The monkey had to detect a change of movement direction, which always happened on the side, not covered by the RF. Stimuli were presented on a 120 Hz CRT monitor. For the presented analysis, all trials were pooled independent of correct or incorrect responses and preferred or anti-preferred orientation outside the RF. The receptive fields were mapped in separate sessions beforehand.

Surgery and recording. Electrophysiological recordings were obtained from six to eight tungsten electrodes positioned in V1. Each electrode's signal was passed through a headstage (Plexon) amplified by a factor of 20. Signal acquisition, filtering, and amplification were done with a Neuralynx Digital Lynx acquisition system. This signal (sampling rate: 32,556 Hz) was used for further analysis.

Analysis. The analysis was done using MATLAB (MathWorks) and the fieldtrip toolbox [54].

Preprocessing. Line noise and the harmonics were removed using the discrete Fourier Transform (dFT) for every analysis except spike detection. Data was padded to 5 s for dft and later cut into trials + /- 1.5 s around stimulus onset. The trial was demeaned, and artifacts were removed by visually inspecting the variance over trial and time. If an artifact was detected, the whole trial was removed. Afterwards we analyzed the 20 sessions with a total of 4863 trials (mean of 243.15 trials per session, SD: 103.52). The data was further analyzed in 6 different ways:

Local field potential (LFP): To get the LFP, data were lowpass filtered (Butterworth IIR filter, zero-phase forward and reverse filter, filter order 6) at 500 Hz.

Multi-unit activity (MUA): For MUA, we followed an approach similar to the one described in [65] and bandpass filtered (Butterworth filter, zero-phase forward and reverse filter, filter order 6) the data between 750 and 8000 Hz. For plotting, we calculated the absolute values of the Hilbert transform of the filtered data.

High frequency power (induced): The high frequency power was assessed using a FFT based time-frequency analysis in combination with a Hanning taper. Frequencies targeted were 20 to 500 in steps of 20 Hz. The frequency analysis was done in shifting time windows (namely over 50 ms in steps of 1 ms). The frequency analysis was done on each single trial; power values were then averaged. Time frequency representation (TFR, Fig. 6**B**) is plotted from 100–500 Hz and baseline corrected (relative baseline change, baseline from −0.6 to −0.1 s) plotted from −0.5 to 0.8 s in steps of 0.025 s. The plotted time refers to the center of the time window.

High-frequency power of the time-domain averaged data (evoked): The same approach was applied for the high frequency assessment of the evoked data; however, trials were first averaged in the time domain (i.e., mean over the trials within one session), and then frequency demodulated in a time resolved fashion (all parameters as described for the non-averaged data).

Gamma power: Gamma power was assessed using the same time-frequency analysis, using a single Hanning taper with the frequency of interest centered at 60 Hz.

Spiking activity: Spikes were detected with a simple threshold approach. They were first highpass filtered at 500 Hz (Butterworth filter, zero-phase forward and reverse filter, filter order 6), and a positive and negative threshold was determined for each recording session separately. A spike was detected at the maximum absolute point crossing the threshold and cut out + /- 2 ms, then plotted to visually check the detection quality. The data was then converted to binary data (with a one at the absolute maximum of the detected spike) and summed over the same time period as used for the other approaches (namely over 50 ms in steps of 1 ms).

The plot of time series: Time-resolved data (i.e., spiking, MUA, LFP, gamma power, induced and evoked high frequency (HF) power, averaged over 400 - 600 Hz) is plotted from −0.2 s to + 0.5 s around stimulus onset for a single session (Fig S11). Data was normalized by (mean-min)/(max-min) to be able to show the temporal relationship between the different measures.

Frequency modulation of high-frequency power: The time-resolved normalized power averaged over 250–450 Hz was frequency demodulated by means of FFT (Hanning window, zero-padded to 3 s). The comparison between HF power during the prestimulus (−0.5 – 0 s) and poststimulus period (0 – 0.5 s) is shown in Fig. 6**B**. The plotted standard error results from the different experimental sessions. Please note that for the model data, the prestimulus period was set to −0.6 to −0.1 and the poststimulus period to −0.1 to 0.4. This was done because there was no delay between stimulus onset and model response. In order to assess the variance, we averaged over 10 consecutive trials for evoked power. We plot the standard error over all 10-trial averages. For the induced power, the standard error is calculated over single trials.

### 2.7 Auditory stimulus encoding and decoding

To test how complex frequency signals are processed in our model, we used the song "Three Little Birds" by Bob Marley https://soundcloud.com/bob-marley-the-wailers/three-little-birds-album, originally released by Tuff Gong on the album Exodus in 1977 and currently licensed by Universal Music Group (UMG). The song was accessed via SoundCloud (https://soundcloud.com/bob-marley-the-wailers/three-little-birds-album) in June 2024. We utilized Python packages, including librosa, soundfile, scipy.signal, scipy.io, and resampy, to perform the following steps:

First, we loaded the song as a.wav file. The original sampling frequency of 44,100 Hz was then downsampled to 1000 Hz to match the model's requirements. This downsampled file was subsequently converted to a.csv file, which indicated the amplitude at millisecond resolution.

Next, we used the .csv file as input for the model at an arbitrary location (x = 0, y = 0), with a signal period set to 3000 ms. We recorded the processed signals at three different locations within the model: (x = 50, y = 0), (x = 0, y = 50), and (x = 50, y = 50).

For analysis, we employed a Short-Time Fourier Transform (STFT) with a Hanning window to examine the original song, the downsampled song, and the processed signals. The window size was set to 128 × 44.1 for the original signal with a sampling frequency of 44,100 Hz and 128 for both the downsampled and processed signals.

Finally, the signals were visualized using the matplotlib.pyplot Python package. This approach allowed us to comprehensively evaluate how complex frequency signals are transformed within the model at different locations. The analysis scripts are provided in the supplementary materials (‘package netlogo/scripts/python’), and the different audio signals are available in the ‘package netlogo/audio’.

### 2.8 Mathematical solution of the network model and encoding/decoding of grey values in images using wave-interference patterns

The mathematical formulation of the network model introduced above can be recast in terms of a difference equation – a discrete analogue of a differential equation – which can be solved to investigate the behavior of the model. It turns out that the difference equations governing the time dependence of the neuronal network model activations are governed by an equation similar to that of damped wave propagation. As we will show shortly, this allows us to mathematically solve the network model: i.e., exactly work out how the network encodes input signals into interference patterns which are the output. These patterns can also be “decoded” using the mathematical structure of the solution; essentially allowing us to calculate the input signal from the resulting interference patterns. We outline the solution and the approach to encoding/decoding here and relegate the detailed description with all its tedious algebraic details to the supplementary material (package netlogo\documents\encoding_decoding_pictures.pdf). The demonstrated picture, adapted from the original image sourced from Discover Wildlife (https://www.discoverwildlife.com/news/theory-of-mind-demonstrated-great-apes), was converted into grayscale, scaled, and resampled to match the input size required by the model.

#### 2.8.1 Notation and difference equations

We will use $A\left(x,t\right)$ to denote the amplitude at node xat time t, making the position dependence of the activations explicit. Similarly, the slope $\Delta \left(t\right)$ at node *x* will be called $\Delta \left(x,t\right)$. We introduce a few more shorthands to ease the presentation of the equations and their solutions in both the main text and the supplement: variable dfor the combination $\left(1+f\right)\left(1+g\right)$, and the damping variables $\delta_{1}=\frac{f}{1+f}$, and $\delta_{2}=\frac{g}{1+g}$. Finally, the grid Laplacian introduced earlier at given node x, $\Delta^{\prime }\left(t\right)=\frac{1}{k}\sum_{\mathrm{Ni}=N1}^{\mathrm{Nk}}\left(A_{\mathrm{Ni}}\left(t-1\right)-A\left(t-1\right)\right)$, will be denoted by $\nabla^{2}\left[A\left(x,t\right)\right]$. The change of activation at a given node, i.e., the difference between the activations at two consecutive time steps is denoted by the difference operator $D_{t}$, a discrete analogue of the derivative, such that $D_{t}\left[A\left(x,t\right)\right]=A\left(x,t+1\right)-A\left(x,t\right)$. Similarly, one has the second order difference operator $D_{t}^{2}\left[A\left(x,t\right)\right]=A\left(x,t+2\right)-2A\left(x,t+1\right)+A\left(x,t\right)$, which is a discrete analogue of the second-order derivative.

In terms of the new symbols, Eq. 1 and Eq. 2 can be recast to give us:

$A\left(x,t+1\right)=\frac{1}{1+g}\left(A\left(x,t\right)+c^{2}\Delta \left(x,t\right)\right)$, and

$$\Delta \left(x,t+1\right)=\frac{1}{1+f}\left(\Delta \left(x,t\right)+c^{2}\nabla^{2}\left[E\left(x,t\right)\right]\right)$$

These equations can be combined to give us a second-order difference equation for time-evolution of the activations given by

(4) $$D_{t}^{2}\left[A\left(x,t\right)\right]+\left(\delta_{1}+\delta_{2}\right)D_{t}\left[A\left(x,t\right)\right]-c^{2}\nabla^{2}\left[A\left(x,t+1\right)\right]+\delta_{1}\delta_{2}A\left(x,t\right)=0$$

The above equation can be solved exactly to obtain how our network model encodes signals and propagates them in time. The solution, detailed in the supplementary material, is easily expressed in terms of $A\left(k,t\right)$ that is the amplitude of the wave with wave-vector kobtained from Fourier-decomposing the real-space signal $A\left(x,t\right)$ into sinusoids with different wave-vectors (or equivalently wavelengths). The key point is that each constituent wave-vector, or each Fourier mode, evolves independently from each other. Each Fourier mode oscillates sinusoidally with a frequency $\omega_{k}$ and is damped (suppressed) in time with factor $\lambda_{k}$:

(5) $$A\left(k,t\right)=\exp\left(-\lambda_{k}t\right)\left(p_{k}\cos\left(\omega_{k}t\right)+q_{k}\sin\left(\omega_{k}t\right)\right)$$

where both $\omega_{k}$ and $\lambda_{k}$ are in general dependent on the wave-vector k, and the explicit k -dependence of $\omega_{k}$ and $\lambda_{k}$ are given in the supplementary material. In fact, $\omega_{k}$ controls the speed of the wavefronts propagating in the network. The solution describes damped propagation of waves on the nodes of the grid. The solution of the problem of nodes coupled with “lateral feedback” corresponds to what is sometimes known as normal mode decomposition; in this problem the Fourier modes $A\left(k,t\right)$ are the normal modes which oscillate with “normal frequencies” $\omega_{k}+i\lambda_{k}$ (see supplement for more details).

#### 2.8.2 Calculations of encoding and decoding of added stimuli

We can consider a protocol where the network is exposed to a stimulus, thus representing the input to the network. In our network model this can be described by a function $v\left(x,t\right)$. Before each step of the modulated main algorithm described above, the stimulus is added to the activation function $A\left(x,t\right)$, modelling the effect of the stimulus on the brain. $v\left(x,t\right)$ can describe both exposure of static stimuli (“image”) for a short amount of time and general time-dependent stimuli (“video”). Before a step of modulated main algorithm at time-step t, we add the stimulus to all nodes xin the network as,

$$A\left(x,t-1\right)=A\left(x,t-1\right)+v\left(x,t\right)$$

and this modified activationis used in the rest of the steps in the update of the main algorithm. Due to this change, the difference equation given in Eq. 1 is changed, and now the “solution,” or in other words the output of the network in terms of the activation values as a function of time, is given by $A\left(x,t\right)=A^{\text{gen}}\left(x,t\right)+A^{\text{part}}\left(x,t\right)$.

$A^{\text{gen}}\left(x,t\right)$ is solution without the stimulus, time-suppressed oscillatory behaviour described by Eq. 5, whereas $A^{\text{part}}\left(x,t\right)$**,** known as the “particular solution,” is particular to the stimulus and can be worked out from it, as detailed in the supplementary material. The solution is also worked out in Fourier-space. A single Fourier component $A^{\text{part}}\left(k,\omega \right)$ of $E^{\text{part}}\left(x,t\right)$ can be worked out only from the Fourier component $v\left(k,\omega \right)$ of the stimulus $v\left(x,t\right)$, independent of other Fourier components. In general, this means if the input stimulus $v\left(x,t\right)$ is specified at all nodes and time steps, one can work out the output $A\left(x,t\right)$ exactly – this is the encoding. Conversely, specifying the output $A\left(x,t\right)$ for all nodes and times allows us to back-calculate the input stimulus $v\left(x,t\right)$ – this is the decoding. If $A\left(x,t\right)$ is not known for all time-steps but only for a grid of time steps, the input $v\left(x,t\right)$ can be decoded for a comparably dense grid of time steps.

When the network is exposed to static image, either for a short time or for a fixed window of time steps, we have a special case – that the input image can be decoded from the output at a given snapshot at any given single time step. Essentially, the stimulus decomposes into functions of space and time, i.e., $v\left(x,t\right)=I\left(x\right)f\left(t\right)$, where $I\left(x\right)$ describes the static image, and $f\left(t\right)$ describes the exposure in time. Essentially the knowledge of $f\left(t\right)$ allows us to decode the static image $I\left(x\right)$ from the output activation$A\left(x,c\right)$ at a single given time $\bar{t}$.

Further details on all methods are given in the **extended methods** in supplementary material.

## 3 Results

### 3.1 Kernel and parameters of the neocortical simulation

There are three central processing steps at the core of the simulation (details in Materials and Methods). The kernel of the model is shown in Eq. 1**,** where *k* indicates the number of neighbors. For each node, we have

(6) $$A\left(t\right)=A\left(t-1\right)+\Delta \left(t-1\right)+\frac{1}{k}\sum_{\mathrm{Ni}=N1}^{\mathrm{Nk}}\left(A_{\mathrm{Ni}}\left(t-1\right)-A\left(t-1\right)\right)$$

(1.) The grid Laplacian Δ' (t) is calculated as the average difference between the activation of node and the activation of its neighboring nodes.

(2.) The actual lateral feedback $\Delta \left(t\right)$ integrates the grid Laplacian over time.

(3.) The final activation $A\left(t\right)$ at the end of a time step is determined by summing up the feedback $\Delta \left(t\right)$ over all times, i.e., integrating the time-integrated grid Laplacian once again.

The three processing steps can be modulated by distinct parameters (here constants) and mimic the feedback between the neuron-like components of the microcircuit (parameters a-g in Fig. 1A). A key parameter is lateral-feedback coupling ($c^{2}$) that modulates lateral feedback received by a node from its neighboring nodes (spatial derivation; see Eq. 1). Lateral amplitude coupling effectively increases the response of a node to excitatory inputs, enhancing the influence of neighboring activations on the node’s state. This represents the strength of excitatory signals, such as those mediated by the neurotransmitter glutamate.

The parameters *f (damping of lateral feedback)* and *g (damping of the activation)* represent neural mechanisms that can dampen the persistent activity of neurons. These inhibitory parameters in the microcircuit represent biological processes like soma-near inhibition by the neurotransmitter GABA. The *damping of* Δ*(t)* (*parameter f*) dampens the speed and relaxation time of the processed signal, and *damping* (*parameter g*) dampens the activation of the simulated neuronal signal over time (Fig. 1**A**). In summary, Eq. 1 (Fig. 1**A**) is transformed into neuronal network (Fig. 1**B**), including the excitatory and inhibitory activity in the microcircuit.

Finally, we transferred neuronal network features such as size, processing speed, timing, and topology into the simulation. For this, 22,500 microcircuits were arranged for parallel processing (Fig. 1**C**, see Materials and methods). For the model, our biological scales are based on the assumption that a microcircuit represents one unit, like a cortical column with a diameter of 500 µm (Markram, 2006, Mountcastle, 1997). Thus, the model reflects a neuronal columnar network of about 60 mm in diameter. For comparison, the human visual cortex was estimated to contain 20,000 columnar processing units (Andrews et al., 1997). In our model, the smallest temporal unit was set to 1 ms to align with empirical observations on wave speed in cortical information distribution [41], [73]. By estimating the distance traveled over time and comparing it to the width of a processing unit (approximately 500 µm), we inferred an approximate processing time of 1 ms. This results in a model sampling frequency of 1000 Hz, which provides a realistic temporal resolution for simulating neural dynamics. The parameter is adaptable and can be adjusted to accommodate different experimental conditions or theoretical considerations.

### 3.2 Interface design of the full-sized model

By design, the model is an input/output interface (Fig. 1**C**). Input signals can be transferred to the model and are coded into activation modulations over time and space. Input signals might represent a neuronal information input, e.g., sensory input or associative input, into the neuronal network. Such input signals interfere with the current information in the model (Fig. 1**C**). Output signals are conceptual analogs of efferent signals, e.g., neuronal activity that would drive an action, like a motor action or a moment of perception (Fig. 1). To read out simulated neuronal activity from the network, we implemented virtual electrodes of different size (white circles in Fig. 1**C**). These virtual electrodes enable extraction of the simulated electrophysiological activity at every unit of the grid. Extraction of the information on its smallest scale is given by the diameter of one microcircuit. In accordance with Mountcastle (1997), the diameter of a virtual electrode was set to 500 µm, representing one processing unit (Fig. 1**D**, electrode 1 – 6). Electrodes can also be arranged to represent a multi electrode array (MEA). For LFP-like signals, the electrode diameter was set to 2 mm and thereby collects the integrative signal from several microcircuits. To mimic EEG-like electrodes, signals of larger areas (e.g., 20 mm in diameter) can be collected (Fig. 1**C**).

Parallel computing was implemented to speed up the information flow between center microcircuits and their neighbors. The oscillating microcircuits were organized in a 2D grid. Of course, this is not the case in a biological situation. For our study, this is a reductionistic architecture in which a given microcircuit is connected with another microcircuit. The microcircuits exist in the brain architecture. While there are recent coupled oscillator network models with high integrative capacity [25], [36], in our highly reductionistic model we only have an array of fields (squares) which transfer the oscillations they receive to the neighbor, following clear rules. The array of squares is an abstraction of an oscillator network model, but we consider the full algorithmic operations on the wave. In summary, this achieves a holographic storage of input information. The connectivity principle can conceptually be seen to represent either short- or long-distance communication. Indeed, despite increasing grid size, the network model is able to show consistent performance due to growing communication overhead (fig. S1).

**Fig. 2:**
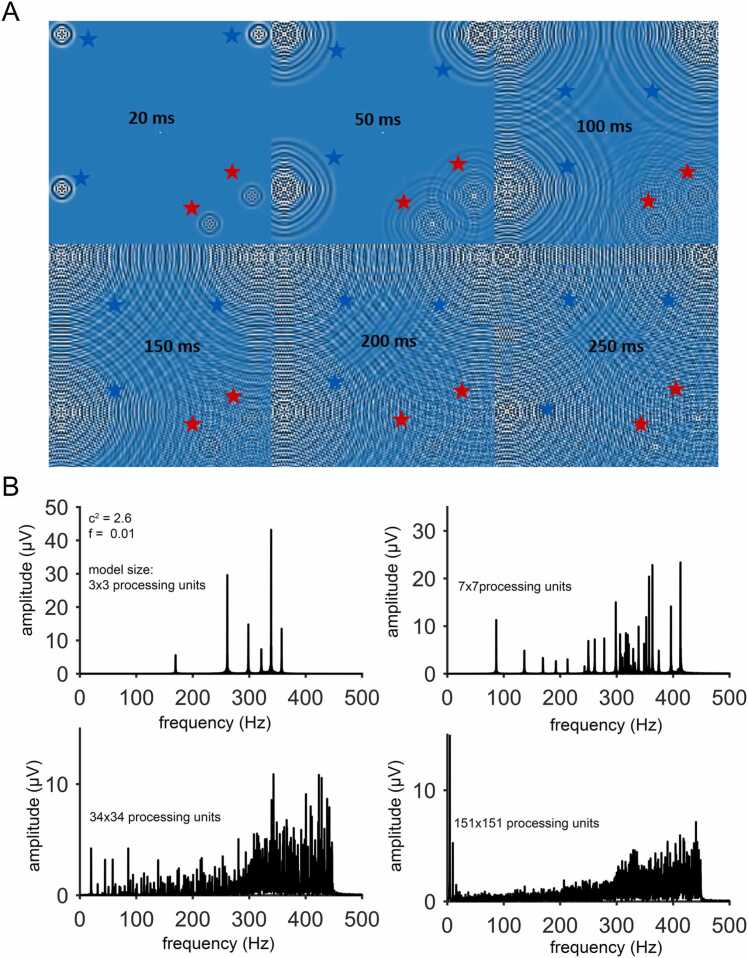
Wave interference patterns, resonances, and model scaling. (A) Waves emerge from the stimuli locations and propagate through the system, eventually interacting and creating complex interference patterns. Six snapshots display the progression of three internal stimuli (green stars) and two external stimuli (red stars) within the simulation at distinct time points (20 ms, 50 ms, 100 ms, 150 ms, 200 ms, and 250 ms). The evolution illustrates how the stimuli are converted into wave-like signals, and how the resulting interference patterns become increasingly intricate over time. For further details, refer to supplementary videos S1-S11. (B) Frequency analysis of the self-organized signals generated by the model reveals stable resonance frequencies across varying model sizes (3 ×3, 7 ×7, 34 ×34, and 151 ×151 processing units). The four sub-panels provide a comparative view of how model size influences the emergence and stability of these resonances.

Neuronal input is simulated by changing the input activation. First, we tested different network input signals with respect to time, peak activation, and space (random, rhythmic, and complex (**video 1**). Excitingly, input signals were convoluted and then spread over the entire model space in a wave-like manner (**video 2,** Fig. 2**A**). This enables the interference of all incoming signals in each unit of the model. This configuration allows the frequency information to propagate uniformly across all nodes in the network, resulting in the representation of information as a unified whole. This behavior aligns with the principles of holographic representation, where each part of the network encodes information about the entire system. Such principles are described in works like Gabor [30] and Bohm [15]. However, we do not want to over-stretch this for holistic models of higher brain processes (e.g. [23], [56]) but be objective and concrete about coding and timing in information processing [21]. We are extending recent efforts on oscillatory models [25] to achieve a powerful yet simplistic computer model demonstrating high integrative capabilities of sensory input, the same information reaches the whole participating neuronal network (this paper, [13] and [12]).

To investigate the integrative capacity of the building principle, the resonance response in frequency space of the signals was analyzed, testing model response with random peak input. An increase in model size (3 ×3, 7 ×7, 34 ×34, 151 ×151 microcircuits) allowed even more stable frequencies to resonate in the model (Fig. 2**B**). This indicates that the model is able to stabilize ('store') activity information increasingly better with growing model size. The true external sensory stimuli are strongly represented by the resonating stable frequencies and their overtones in the model. This suggests that the model can represent external sensory-like stimuli more accurately as its size grows.

In summary, the building principle of this neuronal network allows beyond-local information processing. Information is encoded in time and space by frequency and phase patterns. It is able to integrate high information contents.

### 3.3 Information processing properties of the model architecture

**Fig. 3:**
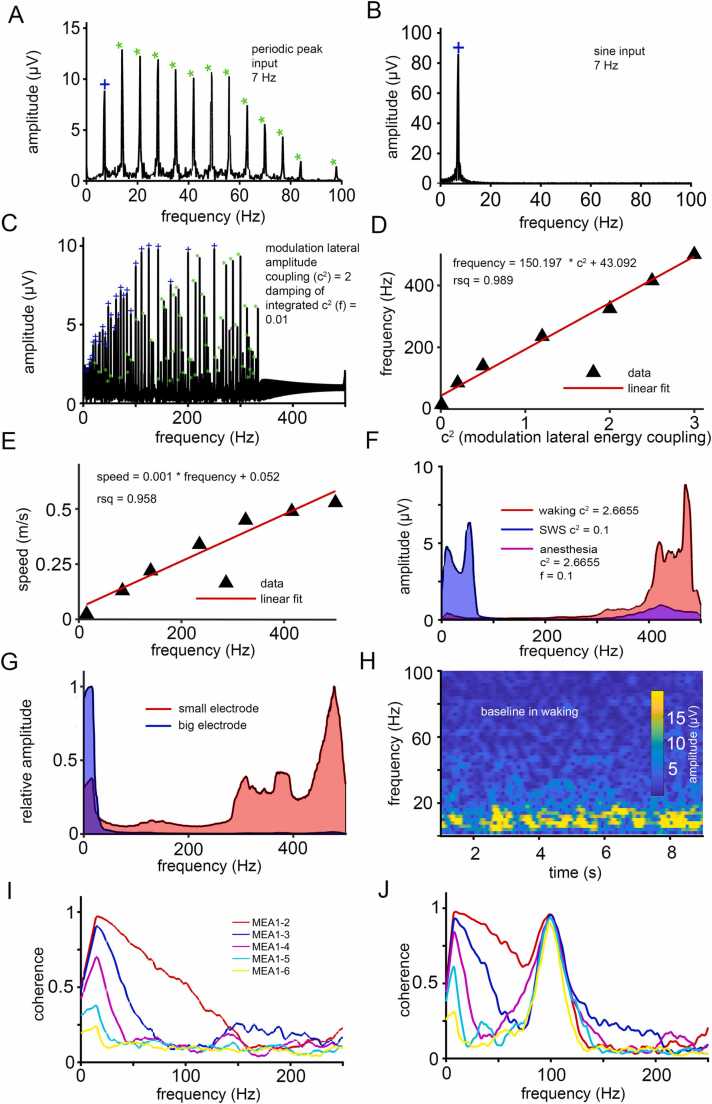
**Properties of the model arising from its wave-like dynamics**. **(A)** Periodic stimulation of the model with a 7 Hz peak input (blue cross) generates harmonic responses (green asterisks), highlighting the system's wave-like nature. (B) In contrast, no harmonic generation is observed when the model is stimulated with a 7 Hz sinusoidal input. (C) Upon stimulation with multiple frequencies, the recorded signal from a single processing unit captures both the input frequencies (blue crosses) and their corresponding harmonics (green asterisks). High-frequency (HF) processing is constrained by a sharp upper limit around ∼330 Hz. (D) This upper limit for HF processing shows a linear correlation with the lateral energy coupling parameter (*c*^*2*^). (E) The velocity of wave propagation positively correlates with the capacity for HF coding, which is itself modulated by the amplitude coupling parameter (*c*^*2*^). (F) The parameters (*c*^*2*^) and (f) together define preferred frequency processing domains, corresponding to distinct activity states of the model that simulate different brain states. (G) HF coding is obscured when larger electrodes are used for recording, while smaller electrodes improve the resolution of HF activity. (H) A frequency-time plot reveals a baseline resonance centered around ∼8 Hz, characteristic of the model's simulated waking state. (I-J) Coherence measures of processing unit activity at varying distances (0.5 mm, 2 mm, 3 mm, 8 mm, 16 mm, and 32 mm) demonstrate frequency-specific signal distribution across the model. This is shown under two conditions: **(I)** when using a chaotic stimulus, and **(J)** with the addition of a 100 Hz sinusoidal signal.

Next, we asked how the model processes signal inputs. We observed the emergence of self-organized harmonics in response to periodic peak signals in the model (Fig. 3**A**). These harmonics decline in the simulation when the signal is sinusoid (Fig. 3**B**). This is reminiscent of activity harmonics that have been observed in in vivo MEA recordings [37].

Many oscillations often co-occur in the same brain state and interact with each other [20]. Therefore, we tested whether our model is able to process several different stimuli in parallel. Using periodic rectangular input (1 ms), the model could resolve more than 50 frequencies (input stimuli and the harmonics) in parallel (Fig. 3**C**) and could offer this information to all participating neurons of the circuit. Having a half-width of the peaks of ∼0.5 Hz within 3 s (fig. S2), the model could code up to ∼329 bit/s in a bandwidth of 7 - 500 Hz. The maximum frequency that can be coded depends positively on the lateral feedback coupling (see Supplementary material) and can reach 500 Hz (Fig. 3**D**). Note that this parameter *c*^*2*^ controls the efficiency of transmission ('synaptic transmission efficacy' (Δ'(t))) between the simulated neurons. In turn, the maximum frequency shows a positive correlation to the speed of the traveling waves (Fig. 3**E**), a biological phenomenon known from electrophysiological recordings of the cortex [73]. For optimal information processing, the model's lateral feedback coupling (*c*^*2*^ parameter) approaches its maximum. This causes emerging resonance in the high frequency (HF) band. As brain rhythms are preserved in mammals and represent certain brain states, we define this state of near-maximal information integration capability of the model as a simulated 'waking state' (Fig. 3**F**, in red).

Based on this starting point, we tested how the model behaves when we modulate its state. To mimic a 'slow wave sleep’-like (SWS) state, we reduced the lateral feedback coupling between neigboured microcircuits. This forced the model to resonate at the low frequency (LF) band (Fig. 3**F**, in blue). Next, we computed a general damping of the network, as it might happen during anesthesia in mammals (Fig. 3**F**, in purple). As expected, measurements with large virtual electrode sizes, meaning collecting the output from multiple microcircuits, caused masking of high-frequency signals (Fig. 3**G**).

Simulated full-spectrum EEG-like recording from the model's waking state indicated a self-organizing baseline at ∼8 Hz (Fig. 3**H**). The LF band decreases to theta activity in the SWS state (fig. S3). Random stimuli given to the model do not propagate over the whole model and coherence declines with increasing frequency and distance (Fig. 3**I**). However, coherence decline in the model can be counteracted by sinusoid harmonic stimuli (e.g., by defined short bursts). By this, signal coherence can persist over larger distances and can stabilize at higher frequencies (Fig. 3**J**).

### 3.4 States of the non-local information processing model

Next, we conducted a deeper investigation of the state transitions within our network. Previous studies have suggested that neuronal correlation networks can reach long-tailed degree distributions, which indicate high information integration, particularly when the system is optimally positioned between random and highly ordered static states.

To test the information integration capability of the model, we analyzed the correlation networks of activation changes across each simulated column over 3000 ms and during short bursts of frequency signals. As shown in Fig. S6A, the degree distributions (*P(k)*) evolve from a short-tailed to a long-tailed distribution as the parameter *c*^*2*^ increases. A long-tailed distribution signifies that the network has transitioned into a state of very high information integration. This transition reflects how increasing *c*^*2*^ – which controls the lateral feedback coupling between nodes – moves the system from a state of low integration to one of high integration. This change is further illustrated in Fig. 3D, which shows how higher values of *c*^*2*^ lead to greater information integration. Additionally, we observed that this pattern becomes more pronounced and stable as the model size increases, reinforcing the notion that high information integration is achieved by maximizing both the lateral feedback couplingy coupling (*c*^*2*^) between nodes and the size of the model.

**Fig. 4:**
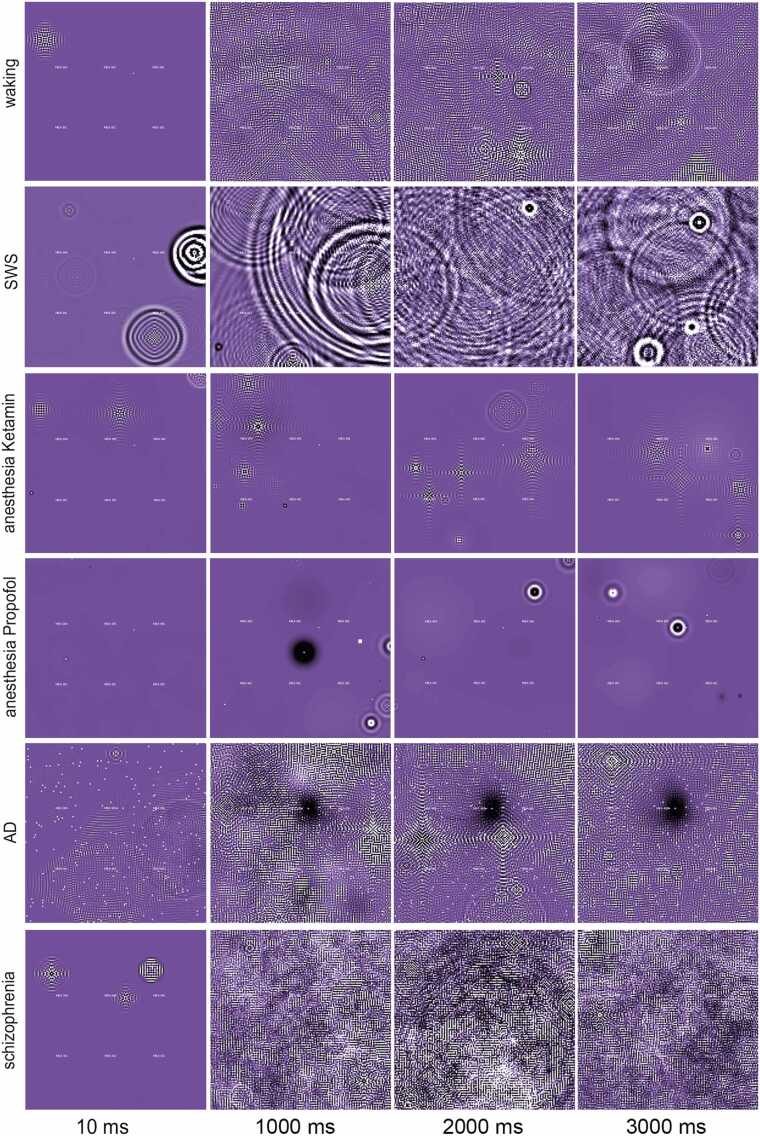
**Video panel of different states of electric activity reflects biological simulations**. By modulating the amplitude transfer within the model (e.g., parameters *c*^*2*^*, f, g*), we can transit between different activity states that we categorize into different simulations of (patho-) physiological states. The screenshots of the visualization of the simulation at different time points give a qualitative impression of the characteristic electric activity of the simulated states. The videos **S1-S11** show the full dynamics, fig. S4 shows the related signals and the parameters for the states are given in Table S1.

By altering the tested parameters for state transition, such as damping and correlation, several different brain states are defined, like the 'waking' state or sleep phases (as indicated in Fig. 3**F**), or describe pathological brain states, like schizophrenia. In our model, examining the influence of the lateral feedback coupling and the different input types on different model states allowed us to investigate the resonance of the model to certain simulated pathophysiological states (Fig. 4 **and** fig. S4**)**. For the input, short information HF bursts superimposed on an LF wave were used to simulate broad and active processing in the brain. When we applied fifty of these complex stimuli, with a random onset at random locations, to the model, a complex non-local interference pattern appeared (**video 4**).

We simulated further network states by fine-tuning model parameters and used the above-described complex input to test those (Fig. 4**;** the corresponding signal simulations, as derived from simulated electrodes, are shown in fig. S4.) Specifically, we reproduced a waking state (**video 4**), SWS (**video 5**), and anesthesia-like state (**video 6 and 7**).

To complement our state analysis, we used Lempel-Ziv Complexity (LZC), a measure of signal complexity based on the compressibility of a sequence, to evaluate the information content of the simulated states. Higher compressibility indicates lower complexity, while less compressible sequences reflect higher complexity. LZC (fig. S6A) allowed us to differentiate between high and low complexity signal processing. Our comparison of states suggests increased information processing during the waking state and a decline in information for the SWS and anesthesia-like states.

We also tested simulations of disease-like states such as Alzheimer's disease (AD; **video 8** and **9**) and schizophrenia (**video 10**). The pathology of AD [29] shows robustness against lesions or defects of neuronal processing units. To simulate lesions, we randomly inactivated single oscillating nodes (representing microcircuits). This caused a high variation of LZC values indicating local differences in complexity of information processing. The simulation could still decode full information when just 4 % of all columns were lesioned. Even when 20 % of the columns were lesioned, information decoding was still possible (fig. S6B). However, information flow between the microcircuit units was interrupted and restricted to local fields (**videos 8** and **9**) (Fig. 4).

Next, we tested what would happen if we lower the correlation of synaptic activitytransfer between the simulated neurons, indicating synaptic transmission defects as those observed in schizophrenia. The synaptic amplitude coupling (*c^2^)* of some of the neighboring columns was randomly impaired causing unsymmetrical processing. As a consequence, the LZC value was dependent on the percentage of uncorrelated coupling between the model units. The 'schizophrenia' model was sensitive to uncorrelated processing as this caused a decline in information decoding. This resulted in the generation of new oscillations that were created by the network but did not correlate with the input (fig. S6C). Moreover, in a stimulus-response task, performed on the schizophrenia simulation, we found a beta-band decline (fig. S6D). Testing of the imbalance between excitation and inhibition within the virtual oscillator increased beta band synchronization in the whole network (fig. S7), and also caused hyperactivity of the network (simulated seizure) (fig. S8).

### 3.5 Encoding and decoding of complex sound stimuli using interference patterns

To evaluate the model's emergent and new ability to process complex frequency signals and offer this information to all participating columns as a basic aspect for higher brain functions, we used the song "Three Little Birds" by Bob Marley, which we downsampled and analyzed at various stages. We asked whether this representative auditory stimulus can be encoded, distributed and successfully retrieved in our array of virtual oscillators.

**Fig. 5:**
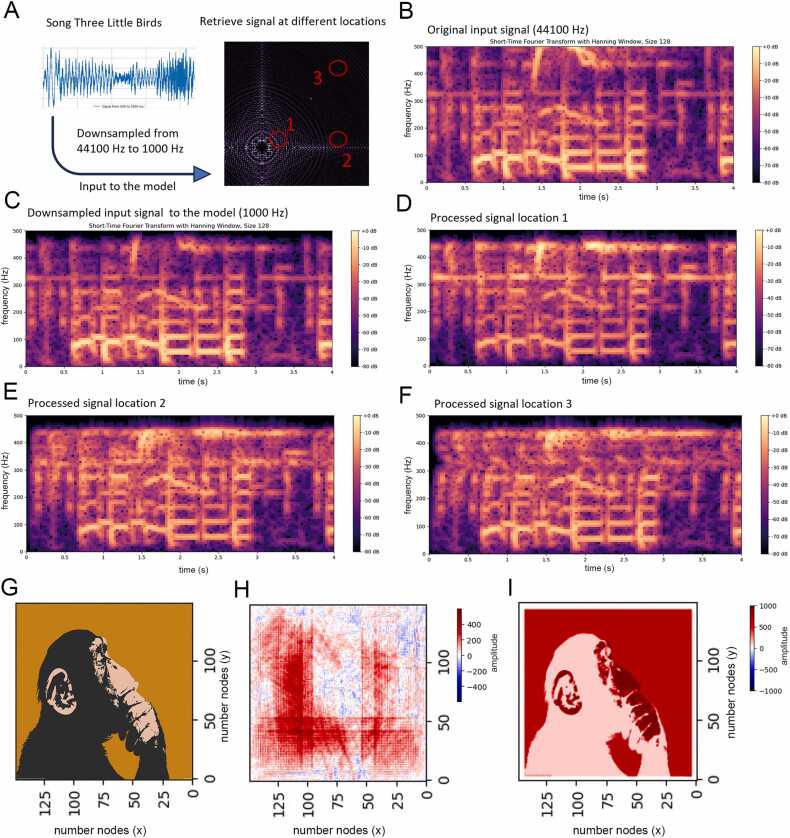
**Encoding and decoding of songs and images using our model**. **(A)** The conceptual overview illustrates the process where a song (signal length 3000 ms) is downsampled and input into the model. The signal is retrieved from three different locations within the model. An STFT is performed to visualize changes in the frequency spectrum over time. **(B)** The original signal is displayed, showing frequency components from 0 to 500 Hz over the entire 3000 ms duration**. (C)** The downsampled signal, maintaining the same time range but with frequencies above 500 Hz filtered out, is shown**. (D-F)** These panels depict the signals processed by the model at the three different locations indicated in (A). Each processed signal is analyzed and visualized using the STFT, demonstrating the model's ability to preserve the frequency identity from 0 to 500 Hz. Corresponding audio (.wav) files for these signals are available in the supplementary materials (package netlogo/audio). (**G**) The process begins with a picture that is converted into grey values. Each pixel's grey value is matched with the corresponding node in the model, ensuring that the pixel position aligns with the node position. The grey values are scaled and mapped topographically into the model. (**H**) The input generates an interference pattern that evolves over time. The figure shows the interference pattern 200 ms after the stimulus onset. This pattern represents the encoded information from the initial image. (**I**) At any point after the stimulus onset, the original image can be decoded from the interference pattern. The example illustrates the image recovery process at various times after onset. The damping effect, which limits the amplitude over time, influences how long the image can be recovered accurately. For small damping factors, the signal decays at a timescale set by ${\left(f+g\right)}^{-1}$. If the evolution time is much larger than this, it is possible that the signal decays to amplitudes which are comparable to machine precision and consequently the loss of accuracy makes the original image unrecoverable. In this example the damping factors are quite small (f∼g∼0.001), and there are no difficulties in recovering the image after approximately 10000 ms.

Using a Short-Time Fourier Transform (STFT), we examined three different signals: the original song (Fig. 5**A**), the downsampled song (Fig. 5**B**), and the signals processed by the model (Fig. 5**C-F**). As expected, the frequency components from 0 to 500 Hz were conserved between the original and downsampled songs. More importantly, these frequency components were also preserved after the model processed the signals. At three different locations within the model (x = 50, y = 0; x = 0, y = 50; x = 50, y = 50), the processed signals retained the original frequency patterns (Fig. 5**D-F**). This demonstrates that the model accurately maintained the integrity of the input signals within the 0 to 500 Hz range across different spatial locations.

### 3.6 Encoding and decoding of image information using interference patterns

Finally, we also tested whether an input representing an image is also successfully encoded and decoded within the interference patterns. This interference pattern contains information about the input image. The process involved converting a picture into grey values, which were then put into the model according to topographic coordinates (Fig. 5**G**). The resulting interference pattern (Fig. 5**H**) evolved over time and space and allowed decoding of the image at various time points (Fig. 5**I**). We observed that a damping effect limited the amplitude over time, but the image could still be recovered, e.g., at 500 and 1000 ms after the stimulus onset. This shows the ability of our neuronal network model to store the integrated complete information transiently.

### 3.7 Direct comparison of model and cortical output using grating stimulation

**Fig. 6:**
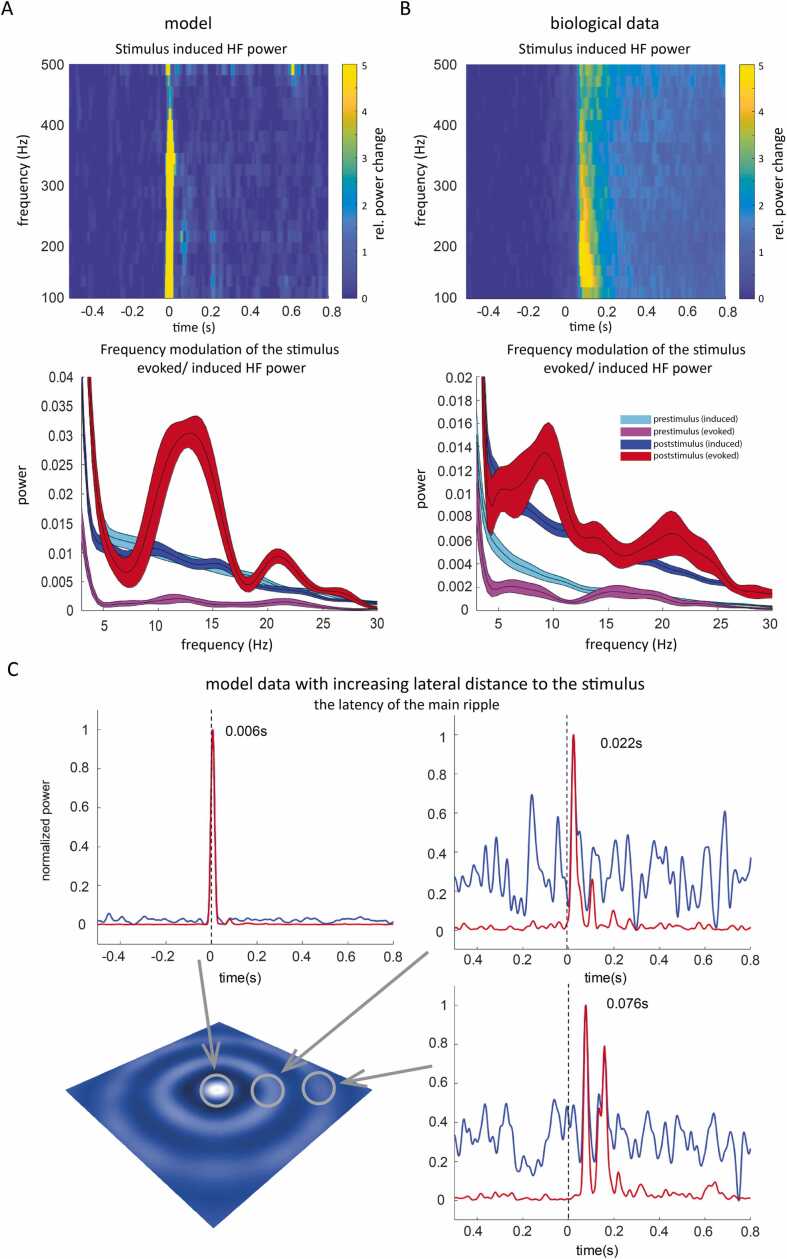
**Stimulus-locked high-frequency activity shows a slow frequency power modulation after stimulus onset in model and biological data**. Analysis of induced and evoked high-frequency activity in microelectrode recordings of macaque V1 neurons responding to visual input (see fig. S9B) and simulated model output to similar input (see fig. S9A). Time-frequency representation (TFR) of induced power is presented: **(A)** for the model and **(B)** from biological data. The lower sub-panels show the temporal modulation of HF power (averaged between 250 and 450 Hz) in a frequency plot. The evoked and the induced power change over time was frequency demodulated (FFT) for a prestimulus (purple and cyan) and poststimulus period (blue and red) of 500 ms. Model and biological-induced HF power show a frequency peak around 10 and 20 Hz, indicating a modulation of HF power at this frequency. This modulation is only found for data after stimulus onset. The power values are normalized for comparison. The colored area depicts the SE over trials. **(C)** This panel shows the temporal evolution of the power (induced - blue and evoked - red) in the 250 - 450 Hz band for simulated electrodes with increasing distance from the stimulus, as shown schematically. The latency of the main power increase is indicated. Time-resolved power for the biological data in comparison to spiking activity, MUA, and gamma power can be found in fig. S11. Induced power corresponds to power measured in the single trial and the averaged over trials. Evoked power refers to the power assessed after averaging over single trials in the time domain, thereby highlighting time-locked changes. TFR is shown with respect to visual stimulus onset (time point 0, relative power change refers to a baseline from −0.6 to

*In silico*, the simulation provides evidence for HF information coding that self-organizes as a traveling wave due to specific resonance properties. If high frequencies are indeed at the basis of in vivo information transmission, this would suggest HF signals in electrophysiological recordings obtained during information processing. We have found such HF signals in various electrophysiological recordings (microelectrode and electrocorticography (ECoG)) in macaque monkeys using a visual task, as described in a preliminary report [31]. We reanalyzed the in vivo V1 recordings during visual stimulation (find the grating stimuli in fig. S9B**)** and compared it to our *in silico* model output in response to comparable input (fig. S9A**; S9B upper paradigm** and **video 11)**. For both biological and model data, we could observe stimulus-induced and evoked broad-band HF power (averaged over 250 – 450 Hz). Evoked activity is defined as the power calculated over the averaged trials, thereby highlighting time-locked modulation **(**Fig. 6 and demonstrated in fig. S10**)**.

If we further assume that HF power depicts a wave-like lateral distribution of information, we should find i) an increasing latency of the stimulus-induced power increase with increasing distance to the stimulus, and ii) a slow temporal modulation of HF power in the vicinity of the stimulated area. Accordingly, we tested for such slow temporal modulation of HF power in V1 microelectrode recordings before and after stimulus onset and compared it to a simulated electrode. Only the evoked HF power showed a slow temporal modulation (in the alpha and beta range) following stimulation onset but not during the prestimulus period (Fig. 6**B**). This was also true for the model data (Fig. 6**A**). We further tested simulated electrodes with increasing lateral distance and confirmed that the latency of the main ripple increased with increasing distance to the stimulus in the model data **(**Fig. 6**C)**.

The biological data further showed that, while the induced HF power largely corresponded to the observed modulation of multi-unit and spiking activity, the evoked HF activity showed a temporal pattern independent from multi-unit activity (MUA), spiking activity, and gamma power (60 Hz) (Fig. S11**)**. Furthermore, the evoked HF activity was distinct from the local field potential (LFP), albeit sharing certain features.

Additionally, in a second data set, which contained ECoG data from the left hemisphere of an additional macaque monkey (please see the supplementary material for details) we plotted the spatial distribution of the evoked and induced HF activity following visual stimulation (Fig. S9B lower paradigm). The ECoG data also showed induced and evoked stimulus-related increases in HF power. The topography of the stimulus-evoked and stimulus-induced HF power further supported the idea of information transfer in a temporally correlated fashion: induced HF power was confined to V1 (fig. S9C), whereas the evoked HF power change readily spread to V2 (fig. S9D).

## 4 Discussion

In the brains of complex organisms, such as mammals and birds, information integration occurs across numerous interconnected processing units (e.g., virtual oscillators, neural assemblies, cortical columns), which together form a coordinated system of brain oscillators that govern neural dynamics [19], [20], [42]. Accordingly, simulated neural correlates are often organized like neural oscillators in excitatory and inhibitory loops, with temporal and spatial recurrent connections [14], [35], [10].

Here, we combine virtual microcircuits with oscillator design in a large-scale computer simulation. Our model simplifies neural oscillators to basic calculations of addition and subtraction of activation, can simulate up to 22,500 interacting processing units (microcircuits), and allows efficient computing. Lateral interconnections and adjustable amplitude coupling constants allow information to be encoded as 'a whole in time and space' by forming one interference pattern, which emerges as traveling waves. The model can predict, mimic, and recapitulate how information, such as electrophysiological, sensory, or motor input, can spread and synchronize over short or long distances in a cortical analog. The model can be used to simulate how electric activity in a neuronal network might affect large-scale information processing between neuronal assemblies or circuits as a basis for higher brain processes.

### 4.1 Background: modeling electric information integration in an artificial neural network

Recent advancements in modeling neural dynamics have highlighted the importance of geometric constraints, oscillatory behavior, and information integration in understanding brain function, placing our modeling effort in the context of stimulating previous work:

The importance of information integration in the physical substrate of the brain has been suggested [66]. The information integration theory was developed to address the problem of explaining the neuronal mechanisms underlying consciousness. Due to its theoretical, philosophical, and mathematical foundations, it is controversially discussed [40]. However, simulations for such information integration in simulated cortical structures tried to explore the integration potential of cortical structures at different simulated network scales [4]. A surprising finding was that cortical structures might reach close-to-maximal information integration by operating close to criticality [5]. Further, Nemirovsky et al. [50] applied IIT to resting-state fMRI, using Phi max to assess information integration in brain networks. In our neural network analog, power-law-like distributions indicate a wide range of coupling modulations that allow stable information integration. This integration is increased by the modulation of lateral feedback coupling, a parameter for synaptic efficiency, and network size, as demonstrated through simulations and derived from mathematical formulations. Decreasing lateral amplitude coupling in network functions acts as a lowpass filter, thus restricting integration of HF content. Increased network size allows the self-organization of more and especially low frequencies. By this, information integration can be precisely controlled and virtually measured. As a side effect of the applied geometric constraints, baseline signals self-organize in our model within ranges (8-10 Hz) consistent with previous findings in cortical recordings [26], [68], [45], and we can observe as well as mathematically conclude an inverse correlation between baseline activity and model size, aligning with similar results reported by Lea-Carnall et al. [38]. Likewise, Likewise, Pang et al. [55] emphasize the role of anatomical constraints in shaping neural oscillations and dynamics, illustrating how brain geometry influences function. Our model introduces tunable coupling constants and model sizes, allowing for dynamic modulation of oscillations, offering a high computational flexibility for simulating brain states and processing of various stimuli not seen in static anatomical frameworks.

In this context, Aggarwal et al. [3] demonstrated how cortical waves coordinate neural assemblies across different brain states, such as wakefulness and dissociative states in mice. While their work focuses on the descriptive properties of these oscillations, our model not only simulates realistic brain states but also aligns closely with actual measured brain states through several analyses. These include baseline activity, wave speed, coherence patterns, favored bandwidth at both microscopic and macroscopic levels, and Lempel-Ziv complexity (LZC). Furthermore, our model provides a robust mathematical framework for understanding different brain states and transitions between them by modulating oscillations. Lastly, its practical application stands out: the model can encode and decode real-world data, such as images and sounds, simulating the impact of oscillatory dynamics on information processing. By bridging hierarchical dynamics, it supports simulations of complex brain functions while providing a precise mathematical basis for realistic brain states. This combination of biological realism, practical utility and robust mathematical foundation underscores the novelty and versatility of our approach.

For example, we use network state transitions to simulate changes in electrical activity under various physiological and pathophysiological conditions, such as wakefulness (conscious), sleep (unconscious), epilepsy (unconscious), anesthesia (unconscious), Alzheimer's disease (impaired), and schizophrenia (impaired). Similar brain states have been simulated in the literature [22]. Like IIT, we use Lempel-Ziv Complexity (LZC) to measure signal complexity, classifying brain states based on their complexity. The LZC values from our model align with reported values for both simulated and measured brain states, distinguishing high-complexity conscious states from low-complexity unconscious states [2], [1], [48], [60]. Our model also provides a mechanistic explanation for these complexity values, offering insights into what they may represent in terms of brain function.

### 4.2 From linear dynamics to the emergence of nonlinear outcomes

Overall, embedding in cortical model literature, as reviewed by Breakspear [17], our model aligns with existing approaches by sharing features with Neural Mass Models (NMMs) and Neural Field Models (NFMs) while offering unique contributions. Both the review [17] and our model emphasize the importance of collective neural dynamics (large-scale oscillatory phenomena) and the brain's ability to integrate information across distributed nodes for understanding perception, movement, and cognition. However, our model places special emphasis on analyzing the direct link between microscopic and macroscopic mechanics, connecting local node interactions with global network oscillations, with non-linear dynamics playing a pivotal role. Unlike traditional models, our approach combines the simplicity of linear dynamics with the emergence of nonlinear outcomes (e.g., resonance catastrophes mimicking seizure waves), providing a versatile tool for simulating the non-linear dynamics underlying brain states while bridging reductionist methods with realistic brain dynamics. Thanks to its reductionist framework, the model is easily scalable (up to 400k units), achieving computational efficiency and enabling real-time simulations of complex phenomena.

Moreover, Hughes et al. [33] demonstrates that wave physics can act as an analog recurrent neural network (RNN) for processing time-varying signals. Our model extends this concept by applying oscillatory dynamics to higher-order brain functions like perception and consciousness, integrating information encoding and decoding for neurophysiological applications.

An important preprint putting our work in context is Effenberger et al. [25]. They focus on the role of frequency and phase in neocortical oscillations for cognitive processing. From this, they propose the abstraction of microcircuit aggregate activity into a single state variable as amplitude of a damped harmonic oscillator and look at wave-based and holistic representations of stimuli including traveling waves (see also [36]). Our model extends this important modelling and considerations (see also [12], [13]) and reveals an emergent pathway from individual oscillators to travelling wave patterns, integrating both incoming sensory inputs and internal information, and distributing it across all participating nodes (holographic representation).

Unlike Hughes et al. [33], focused on signal processing, and Effenberger et al. [25], centered on cognition, our model investigates oscillatory dynamics' role in higher brain functions. It demonstrates that wave-based mechanisms are not only efficient for information encoding and decoding but also crucial for explaining complex neurophysiological phenomena related to conscious perception.

### 4.3 High correlation with neurobiological observations upon signaling

In addition, our model captures several additional biological phenomena described in the literature. These include traveling waves [49], harmonics [37], parallel processing of multiple frequencies [20], [52], correlations between frequency and propagation speed [73], frequency-specific resonances [22], and frequency-distance-coherence [44], [63]. With all these characteristics, the model addresses concepts of population synchronization and frequency coding.

To validate the alignment between our model’s phenomena and observed electrophysiological activity in the cortex, and to test its applicability in simulating complex neural signals related to perception, we directly correlated simulated outcomes with biological data from visual stimulus-induced and evoked potentials. Our focus was particularly on cross-frequency coupling and high-frequency activity, as observed in neurobiological responses to explicit stimuli. Notably, increasing the lateral feedback coupling efficiency in our model allows high-frequency coding. This is interesting because it is able to simulate properties in our model with higher order function of the brain, as observed during conscious perception in humans and monkeys. In neurobiology, HF activity is typically captured as multi-unit activity (MUA), a neural correlate of spiking activity [18], [47], and can show an inter-areal phase coupling between task-relevant areas e.g., in a visuo-motor task [9]. In order to test our hypothesis and see if the information integration proposed by our model indeed reflects neocortical brain activity, we looked at electrophysiological data of a perceptual task in the primate model. We found that the time-locked HF output of the visual cortex was ordered by a slow phase pattern. We propose that the observed coupling between a high-frequency, input-locked signal and a low-frequency phase might form a fundamental core of neural activity modulation representing lateral and vertical information flow within and between cortical areas.

Our model captures the dynamic distribution, integration, and encoding of neuronal information through the continuous forwarding of frequency and phase data. It offers a conceptual visualization of how electrical activity might be integrated across both temporal and spatial domains.

## 5 Limitations

Our reductionistic model has clear limitations such as (i) lacking explicit memory functions, (ii) actual stimulus recognition happens in higher level resources within the brain columns. We use our networks as "reservoirs" in the machine-learning sense – this means that they are used as a filter that transforms inputs into some other representation using a pre-defined and fixed connectivity. This is in contrast to the aforementioned publications which also study learning in such models.

However, the model currently lacks a biological-analog layer for the decoding of oscillatory patterns, which is achieved here through methods such as Fourier transformation. One could imagine a similar mechanism occurring in cortical layers responsible for decoding and learning. In this context, while the adjustable coupling constants provide flexibility, the absence of learning mechanisms such as synaptic plasticity restricts the model's capacity to simulate adaptive processes in its present form. Nevertheless, these coupling constants can be modified to adapt to specific signal processes.

Additionally, the model’s simplified 2D grid topology may limit its ability to fully capture the complexity of 3D neural circuits and detailed cellular mechanisms, such as synaptic plasticity and ion channel dynamics. While this simplicity enhances flexibility and scalability, it may reduce biological realism by focusing on broad oscillatory behavior and network-level interactions, potentially overlooking finer neurobiological details. Despite these simplifications, the model still facilitates surprisingly accurate comparisons with stimulus-evoked potentials.

### 5.1 Beyond local processing in traveling waves as an emergent path to higher brain functions

Convergent evolution in different organism groups (mammals, birds, and maybe others) is a striking argument that interconnected, oscillating microcircuits allow beyond local information processing and are a physical substrate important for higher brain function [20], [62]. An example for convergent evolution is the columnar architecture of the neopallium of birds which has been proven to be suited for processing perceptual and cognitive abilities [51], [64].

Our model shows an emergent path from individual oscillators and wave patterns to a highly efficient central circuit integrating all information, incoming, like a sensory input, or outgoing, like activity during perception and sharing it completely with all participating columns. Additional emergent levels are achieved by increasing the number of processing units (here, local microcircuits) that increase the stable resonances for frequencies. This adds a growing number of frequencies that are processed in parallel, allowing unit by unit a more complex and stable representation of information. One might argue that in this way, a sufficient size is soon reached that is necessary for global environmental information processing. The model supports the traveling wave concept as well as the concept of beyond-local information processing in a central circuit. If these turn out to be correct, this might be an important basis for higher brain function. Cognitive functions such as perception, and maybe even consciousness, may fundamentally emerge from the holistic integration and presentation of information as shared wave patterns, synchronizing activity across multiple rhythms within central brain circuits.

## 6 Conclusion

Extending and building on previous integrative oscillatory neuronal models, we show a here a reductionistic, integrative neuronal network that can compute a variety of cortical electrophysiological phenomena in one model environment, but also show practical applicability to encode, transiently store, integrate and decode sensory information (images, music). Even minor modulations of the lateral feedback coupling between and within neuronal oscillators allow for logical transitions between model states. The parameters are explainable and biologically interpretable. Thus, our work provides computer codes for an efficient way to process, share with all CPUs, distribute, and integrate the same complex information. These principles might be interesting not only for future powerful computer systems, but also for testing neurobiological hypotheses or for decoding of ‘real’ electrophysiological data (local field potential, EEG, ECoG, MEG, and others, considering also the high frequency part) from the nervous system of different organisms.

## Funding

This work was supported by the 10.13039/100004807DFG (Project number 492620490 - SFB 1583/INF [to TD; signaling aspects]) and the Land Bavaria for funding (contribution to DFG project 324392634/TRR 221-INF [to TD; software, modelling]). BH was funded by an 10.13039/100010663ERC starting grant (#677819). RB was funded by the 10.13039/100004807DFG, project 424778381/TRR295 project A02.

## CRediT authorship contribution statement

**Johannes Balkenhol:** Writing – review & editing, Writing – original draft, Visualization, Validation, Software, Methodology, Investigation, Funding acquisition, Formal analysis, Data curation, Conceptualization. **Barbara Händel:** Writing – review & editing, Visualization, Validation, Methodology, Investigation, Funding acquisition, Formal analysis, Data curation, Conceptualization. **Sounak Biswas:** Writing – review & editing, Methodology, Investigation, Formal analysis. **Johannes Grohmann:** Writing – review & editing, Software, Resources, Methodology, Investigation. **Jóakim v Kistowski:** Writing – review & editing, Software, Resources, Methodology, Investigation. **Juan Prada:** Writing – review & editing, Software, Methodology, Investigation, Formal analysis. **Conrado A. Bosman:** Writing – review & editing, Validation, Methodology. **Hannelore Ehrenreich:** Writing – review & editing, Investigation, Formal analysis. **Sonja M. Wojcik:** Writing – review & editing, Validation, Resources, Methodology, Investigation. **Samuel Kounev:** Writing – review & editing, Supervision, Software, Resources, Methodology, Investigation. **Robert Blum:** Writing – review & editing, Supervision, Resources, Project administration, Methodology, Investigation, Funding acquisition, Formal analysis, conceptualization. **Thomas Dandekar:** Writing – review & editing, Writing – original draft, Supervision, Resources, Project administration, Methodology, Investigation, Funding acquisition, Formal analysis, Conceptualization.

## Data Availability

All data supporting this study are included in the manuscript, its figures, and the supplementary materials. This includes links to download the complete program code, along with a tutorial for its use, available on our homepage at https://www.biozentrum.uni-wuerzburg.de/bioinfo/computing/neuro and on GitHub at https://github.com/Department-of-Bioinformatics/non-local_cortex_simulation. Also, the code used to process the animal data and to obtain the shown data figures is made fully available. We allow for data redistribution for the purpose of replication. Data availability statement – monkey data: The monkey data used for testing the model predictions were data originally recorded for a different purpose and research question. The data therefore is in possession of the recording institute (Donders Institute, Nijmegen, Netherlands) and is administered by Pascal Fries. A set of publications has been published based on this data set (1−8) and the data have been made available as described in these publications.
