# Supplementary material for: Beyond-local neural information processing in neuronal networks
Source: Comput Struct Biotechnol J. 2024 Nov 13;23:4288–305. doi: 10.1016/j.csbj.2024.10.040 (PMC11647244; doi:10.1016/j.csbj.2024.10.040)
Supplement: Supplementary file 2 — Supplementary material [file mmc2.pdf]

Supplementary material:

Beyond-local neural information processing in neuronal networks

Johannes Balkenhol [1,§], Barbara Händel [2,7§], Sounak Biswas [9], Johannes Grohmann [5], Jóakim v. Kistowski [5], Juan Prada [1], Conrado A. Bosman [3], Hannelore Ehrenreich[4], Sonja M. Wojcik [6], Samuel Kounev [5], Robert Blum [7,*], Thomas Dandekar [1,8,*]

[1] Department of Bioinformatics, Biocenter, University of Würzburg, 97074 Würzburg, Germany

[2] Department of Psychology (III), University of Würzburg, 97070 Würzburg, Germany

[3] Cognitive and Systems Neuroscience Group, Swammerdam Institute for Life Sciences, Center for Neuroscience, University of Amsterdam, 1105 BA Amsterdam, Netherlands

[4] Experimentelle Medizin, Zentralinstitut für Seelische Gesundheit, 68159 Mannheim

[5] Institute of Computer Science, Chair of Software Engineering (Computer Science II), University of Würzburg, 97074 Würzburg, Germany

[6] Neurosciences, Max-Planck-Institut für Multidisziplinäre Naturwissenschaften, 37075 Göttingen; Germany

[7] Department of Neurology, University Hospital Würzburg, 97080 Würzburg, Germany

[8] European Molecular Biology Laboratory (EMBL), 69012 Heidelberg, Germany

[9]Department of Theoretical Physics I, University of Würzburg, 97074 Würzburg, Germany

[§] Authors contributed equally

[*] Corresponding authors: dandekar@biozentrum.uni-wuerzburg.de, blum_r@ukw.de

Content

Supplementary Figures 3
Figure S1: Computational demand of the proposed model. 3
Figure S2: The half width of the peaks indicates a mean half-width of around ~0.5 Hz within 3 s. . 4
Figure S3: Simulated arousal: Basal brain EEG activity changes from theta (SWS) to alpha activity (waking). 4
Figure S4: Signals recorded from small and large virtual electrodes at different model states. 5
Fig S5: Distribution chnages form short to long tailed distribution indicate state changes form low to high performance processing. 6
Figure S6: Neurophysiological states comparing model and observations. 7
Figure S7: EEG beta firing increases with increased relative activity of inhibitory neurons 8
Figure S8: Analyzing epilepsy considering the critical borders of information processing............ 9
Figure S9: In silico and in vivo stimulus paradigm. 10
Figure S10: Revealing the stimulus locked signaling after grating stimulation in the simulation. . 11
Figure S11: Time-resolved modulation of various neural signals obtained from microelectrode recordings of macaque V1. 12
Fig S12: Dependance of oscillation frequency and damping on the wavevector (wavelength)...... 13
Table S1: Summary of model parameters and their values for simulations in this study. 14
Extended methods 17
Microelectrode recording in macaque monkey 17
Behavioral paradigm and visual stimulation. 17
Surgery and recording 17
ECoG recordings in macaque monkeys 17
Visual Stimulation and Attention Paradigm 17
Surgery and recording 18
Details of Mathematical Solution of the Network Model 18
Additional online information and data visualization 22
Supplementary references 23

Supplementary Figures

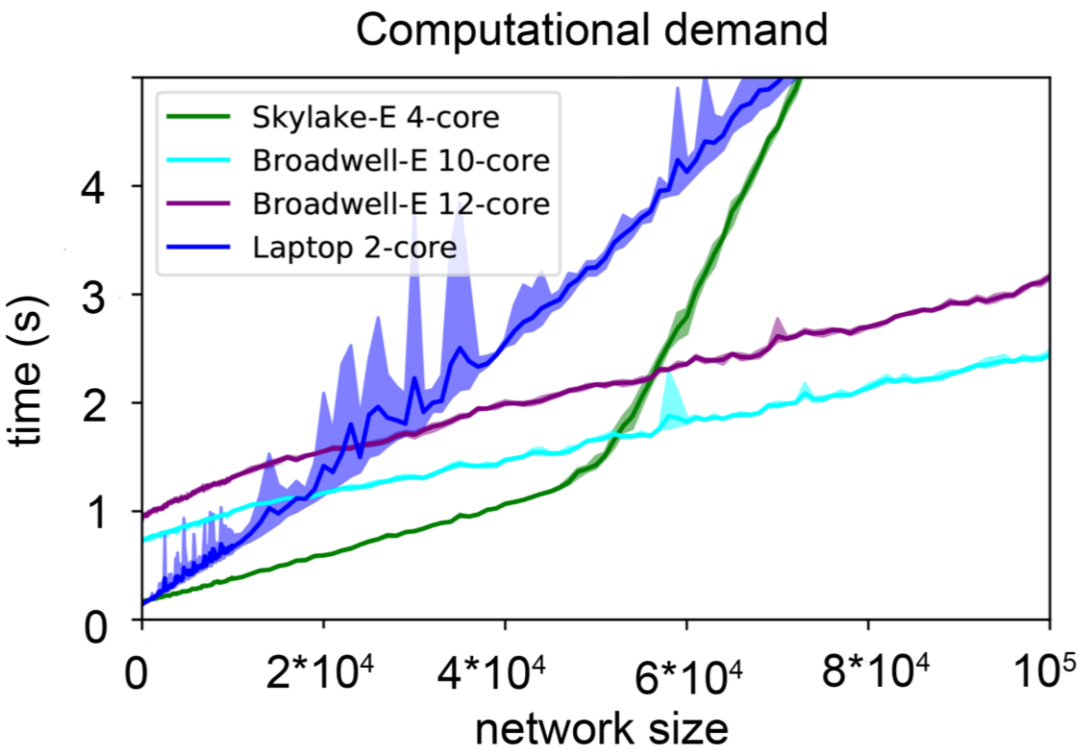

Figure S1: Computational demand of the proposed model. The computational performance of the neural field model, emphasizing its suitability for parallelization due to the interconnected architecture. The simulation was run on different computer architectures, highlighting the impact of grid size (number of simulated nodes) on performance. The scalability of the model was tested on a variety of core technologies, including Skylake-E 4-core, Broadwell-E 10-core, and others. The results demonstrate how the computational demand scales with the number of cores and nodes, showcasing the model's potential for efficient execution on high-performance computing clusters. Certainly, supercomputing can go up orders of magnitudes higher, but we have here a clear upscaling of several orders of magnitude compared to the initial model. Moreover and interestingly, despite this upscaling by several orders of magnitude, no new higher order phenomena emerged, this is our point.

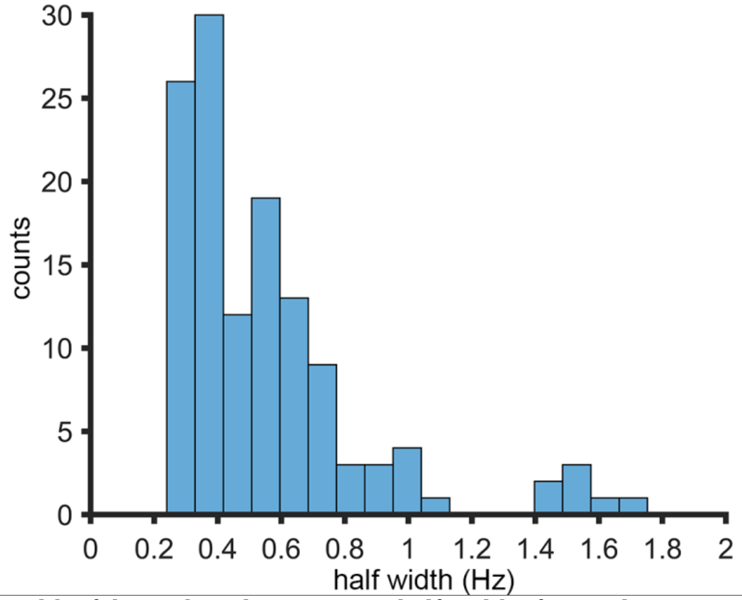

Figure S2: The half width of the peaks indicates a mean half-width of around ~0.5 Hz within 3 s. These are the results from the peak analysis of the simulation of prime number frequency input from 2-239 Hz, the half-width of the peaks is demonstrated. The input frequencies were continuously applied. The length of the analyzed signal was 3,000 ms. The *NI_slopev* is 2.6 and the *slopeo_damping* is 0.01. The peak analysis shows that non-local information storage is efficient and works over the whole bandwidth. Using the equally distributed half-width of the peaks, the coding potential within a distinct time frame can be estimated (~329 bit/s in a bandwidth of 7-500 Hz at a single location). The determination of the peaks becomes technically easier by dividing the frequency space through its own running average (window size = 60 data points).

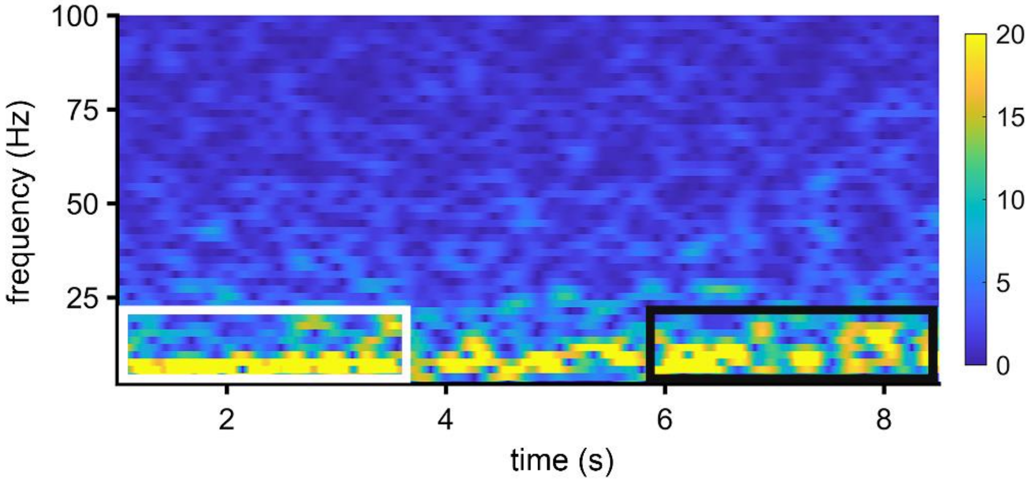

Figure S3: Simulated arousal: Basal brain EEG activity changes from theta (SWS) to alpha activity (waking). Baseline changes are mediated in our model by changes in the parameter c^2 (theta to alpha). Here, the *NI_slopev* was stepwise increased from 0.1 to 2.8 by 0.3 each 1 s. The transition from theta (white box) in the SWS states to dominating alpha activity (black box) in the waking state has been observed in EEG recordings in men [1]. The parameters were the same as in **Fig. 3H**.

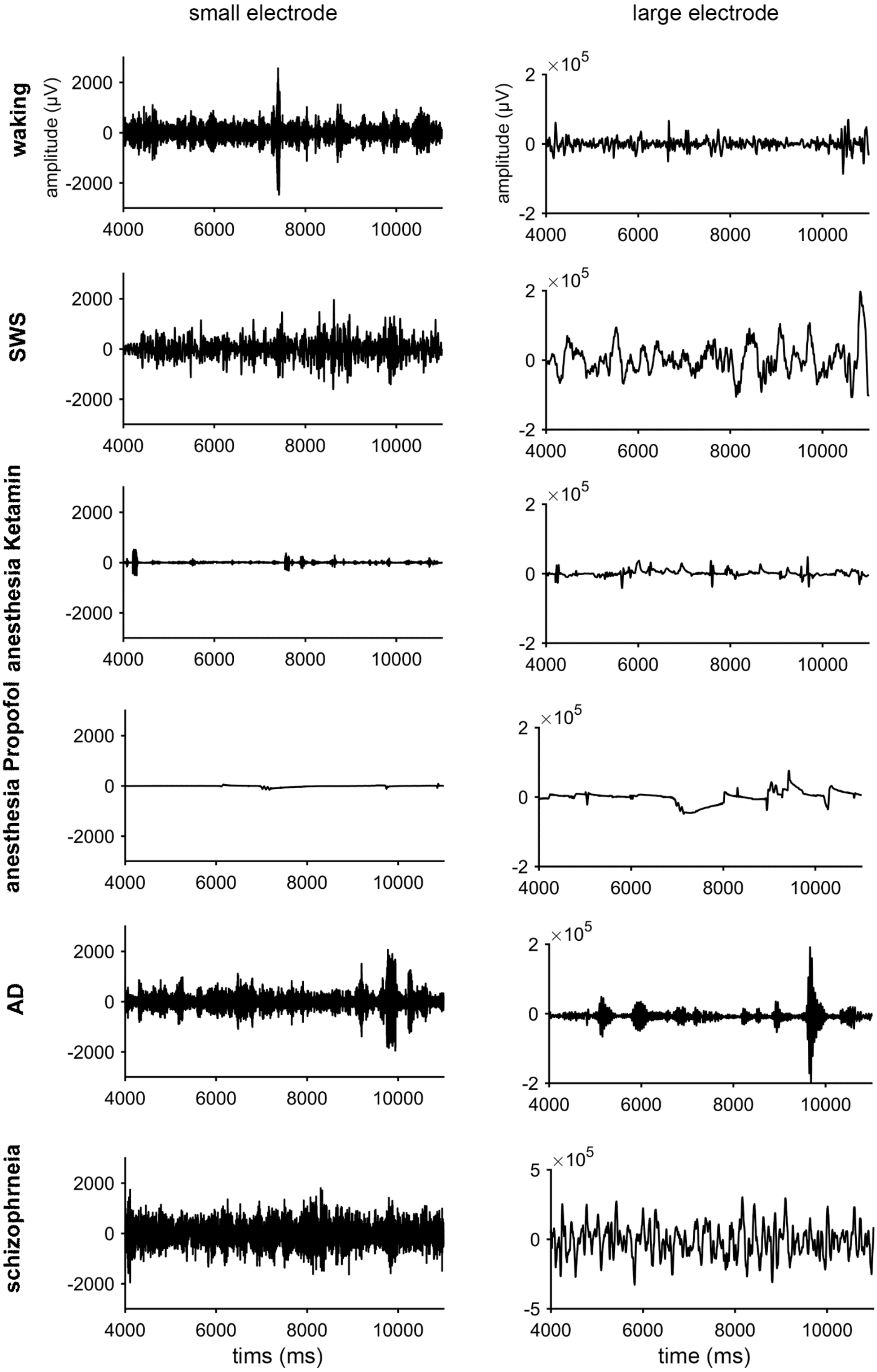

Figure S4: Signals recorded from small and large virtual electrodes at different model states. The signals correspond to the simulations shown in **Fig. 4**.

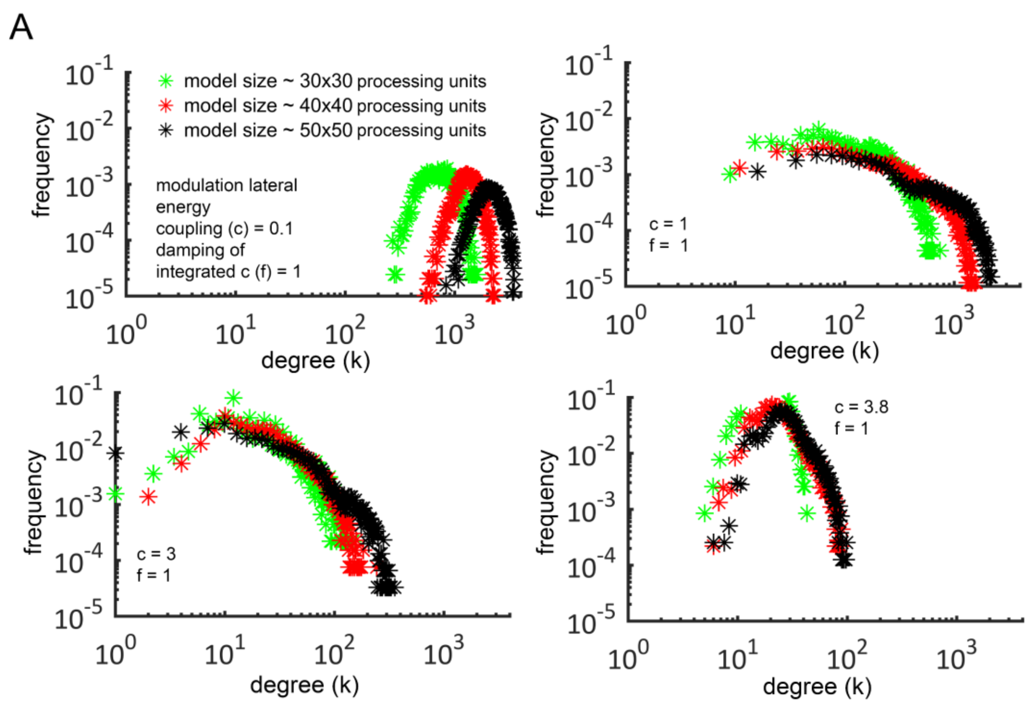

<u>Fig S5: Distribution chnages form short to long tailed distribution indicate state changes form low to high performance processing.</u> (Color) degree distribution for positively correlated networks. The four sub-panels depict the degree distribution for the correlation networks at different values of lateral amplitude coupling c^2=0.1, c^2=1, c^2=3, c^2=3.8, for three representative model sizes 30x30, 40x40, and 50x50 processing units.

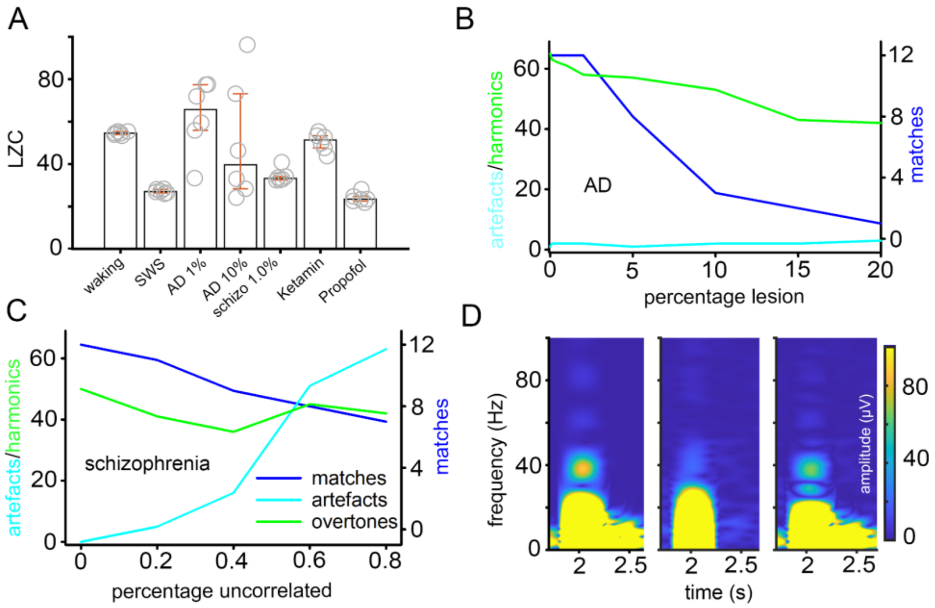

Figure S6: Neurophysiological states comparing model and observations. **(A)** The Lempel Ziv complexity (LZC) can be used to quantify the discrimination of the model states shown in **Fig. 4** by evaluating the complexity of information processing. LZC values are stated as the median of six electrodes and error bars indicate the 25% and 75% quantile. **(B)** The decline of information processing by the increase in the number of lesions in the model is evaluated by the number of input frequencies that could be resolved (matches), the number of related overtones that organize (harmonics), and the number of input-unrelated frequencies (artefacts). **(C)** The effect of uncorrelated amplitude coupling in the model (schizophrenia) is evaluated. **(D)** A model adjusted for the neurophysiological lower correlation in a model of schizophrenia. We analyze here the gamma-band decline during stimulus processing. The self-organizing gamma-band following a stimulus is shown for a simulated healthy neocortical model (left) and simulated schizophrenia (middle). The difference between correlated (waking) and uncorrelated processing is shown on right. The color bar indicates simulated activity in µV.

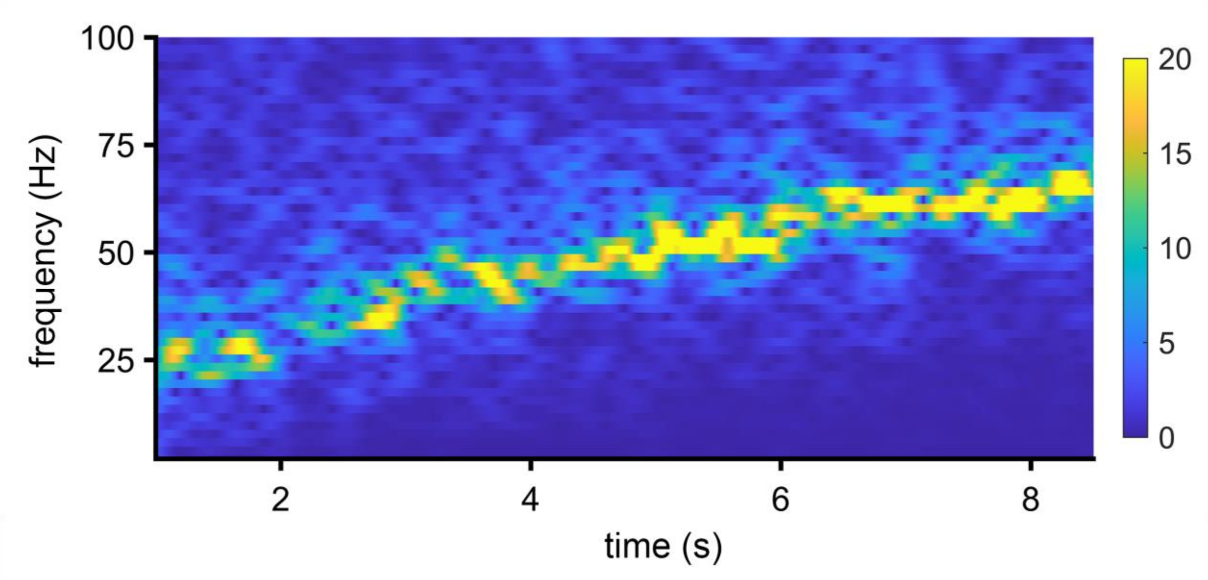

Figure S7: EEG beta firing increases with increased relative activity of inhibitory neurons. The increase of inhibitory activity by increasing the *ratio_inhibition_activation1* is displayed. The *ratio_neighbour_activation* (excitatory neurons) and the *ratio_inhibition_activation1* (inhibitory neurons) were set to 0.8 at time point 0. The *ratio_inhibition_activation1* is increased each second by 0.02. As a result, the self-organized frequency band is increasing gradually. This illustrates that the resonating frequency band increases due to more inhibitory neural activity in our simulation.

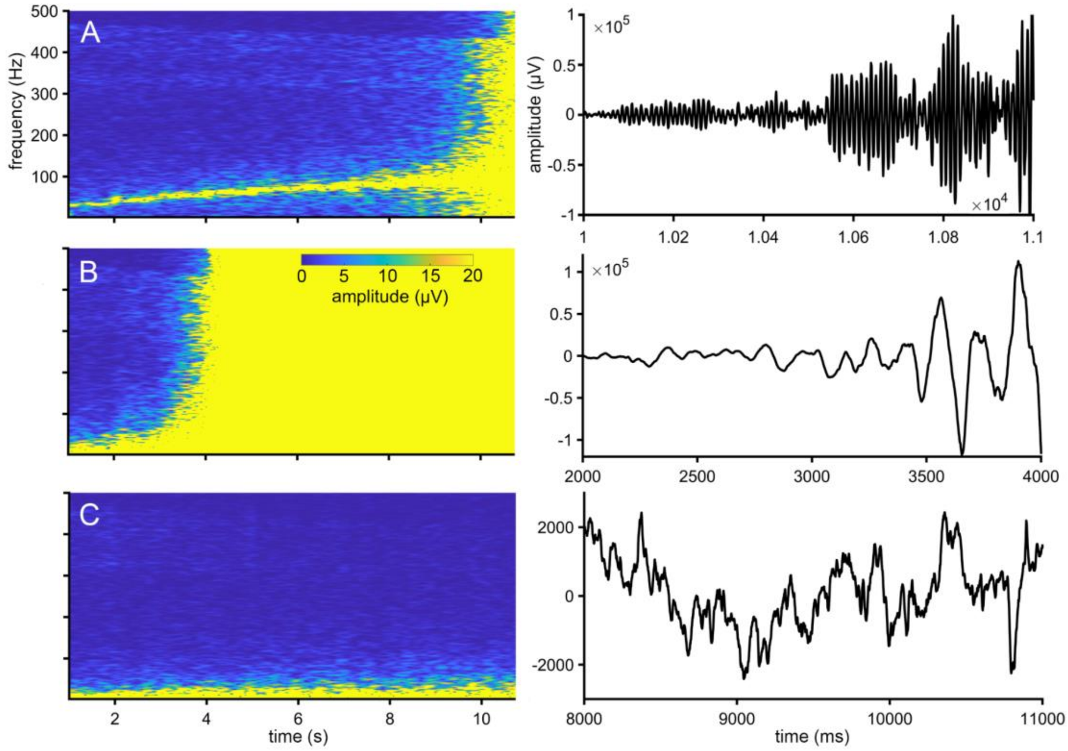

Figure S8: Analyzing epilepsy considering the critical borders of information processing. System collapse can have many reasons. To investigate the origins of epilepsia the amplitude coupling parameters were systematically altered. Especially an imbalance of excitatory and inhibitory neurons and uncorrelated processing can impair information processing and can cause energies to escalate. The system behavior was investigated when processing at different integration steps was uncorrelated and unsymmetric. **(A)** The *ratio_transmitted_amplitude* of the neighboring neurons was gradually decreased by 0.05 until it reached 0.5 (increased activatory and decreased inhibitory influence). The process of declining the *ratio_transmitted_amplitude* was uncorrelated (modulated by a random decline between 1 and 0.5). The amplitude coupling of **(B)** *slope_vector* and of **(C)** *slope_old* was decreased similarly (mathematical procedure as described in (A)). An uncorrelated change in the extent of *slope_vector* and *slope_old* was not disrupting the balance of excitatory and inhibitory neurons, but the balance of integration steps. This could lead to the escalation of signals as well **(B,C).**

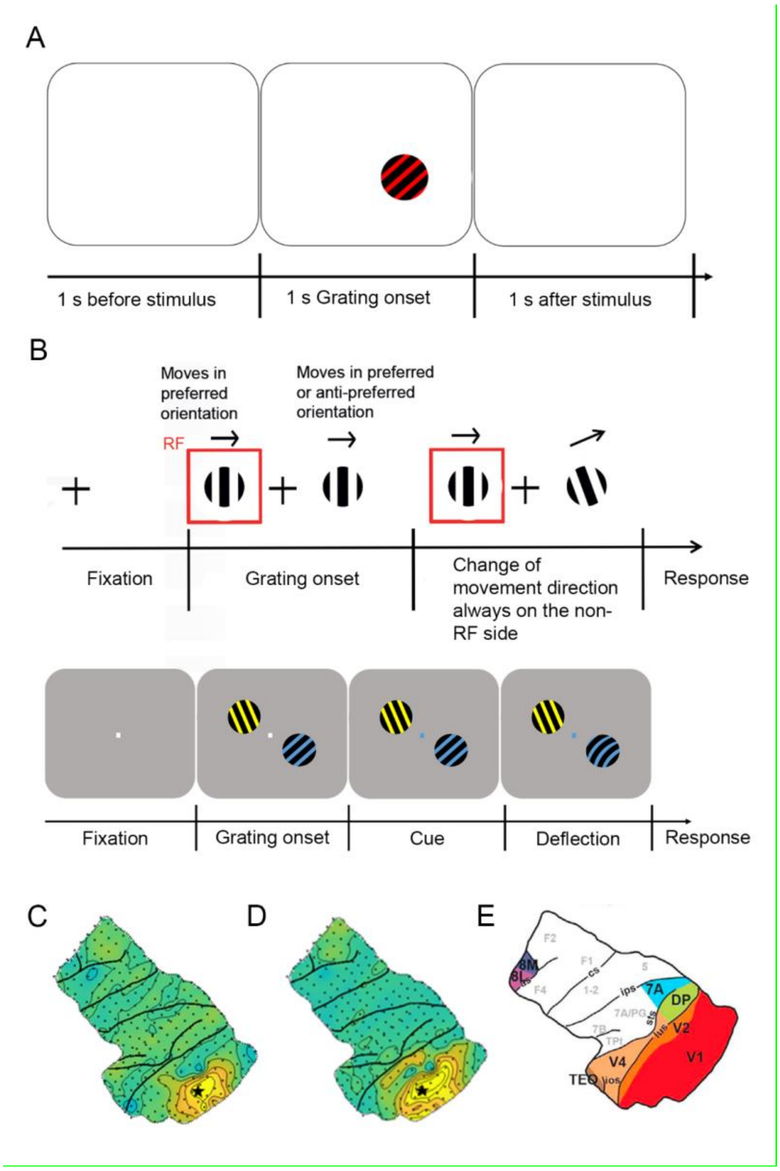

Figure S9: In silico and in vivo stimulus paradigm. **(A)** The parameters of the grating and the simulated electrodes were chosen to be close to the in vivo experiments. The grating was defined by a harmonic stripe pattern introduced by constant positive (+10 mV, shown in red) and negative (-9 mV, in shown in black) activation levels. The input was permanent, as long as the grating stimuli was on (1 s) and was turned off for 1 s before and after the grating. For the simulation, data was calculated for a small and a big virtual EEG-electrode (radius = 2.5 mm and 10 mm). The small proximate electrode measured direct sensory input at the site of the grating (distance = 0), thus fully overlapping with the stimulus. The larger distant electrode measured at a distance of 15 mm to the stimulus center, thus partly overlapping with the stimulus. The model size was 75x75 mm. Other model parameters were the same as for the simulated waking state (see M&M). **(B) Upper paradigm** as used in the V1 microelectrode recordings. A fixation point was presented during a prestimulus baseline (1 s), followed by two luminance gratings (contrast of 100%, diameter of 2–3°, spatial frequency of 1–2 cycles/°, temporal frequency of 1–2°/s). The grating within the receptive field (RF) of the recorded neurons always had the preferred orientation and was moving. The grating outside the RF, on the opposite side of the fixation point, was either presented in the preferred or anti-preferred orientation and was also moving. The monkey had to detect a change of movement direction, which always happened on the side, which was not covered by the RF. **Lower paradigm** used in the

ECoG recordings. A prestimulus baseline (0.8 s, fixation) was followed by the presentation of two isoluminant and isoeccentric drifting sinusoidal gratings (diameter: 3°, spatial frequency: ≈1 cycle/deg, drift velocity: ≈1 deg/s, resulting temporal frequency: ≈1 cycle/s, contrast: 100%), one yellow, the other blue. After 0.8–1.3 s, the fixation point changed color indicating which grating was task-relevant. At random time points between stimulus onset and 4.5 s after cue onset, either the cued or the uncued grating was slightly bent and the monkey should release the bar on detection of the change.

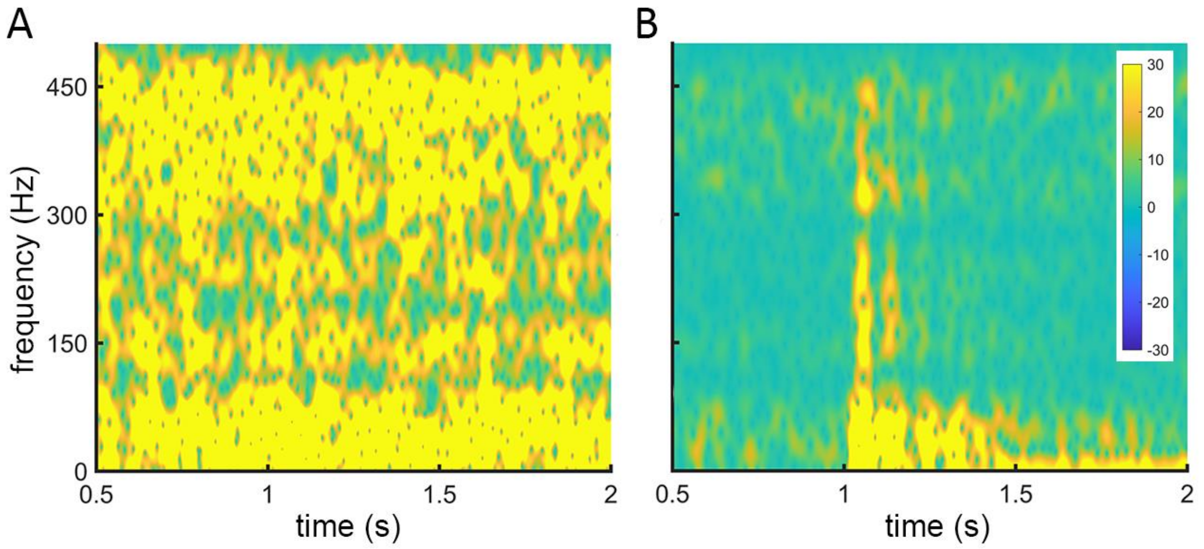

Figure S10: Revealing the stimulus locked signaling after grating stimulation in the simulation. We demonstrate here the simulated grating signal response with a short-time Fourier transform. The onset of the grating signal was 1 s after the trial start. The grating signal lasted for 1 s and a period of 1 s followed after the offset of the grating signal (here only 2 s outlined). The trial was repeated 100 times resulting in a signal length of 300 s. 21 short-burst signals per second of random HF and LF coupling, random onset, and random frequency were applied to the model to simulate an active background processing model. Decoding the signal of a single trial at a distant stimulus (30 mm from the stimulus center) showed a highly loaded coding in frequency space, the **induced potential (A)**. In comparison, 100 trial signals were averaged in time and subsequently transformed to frequency space. Stable encoded frequency in time and space was still recognized after the averaging whereas signals with random onset and random location showed interference depletion, the **evoked potential (B)**. It can be noted that constant non-periodic input, such as the grating signal, induced periodic HF and coupled LF coding in the non-local architecture.

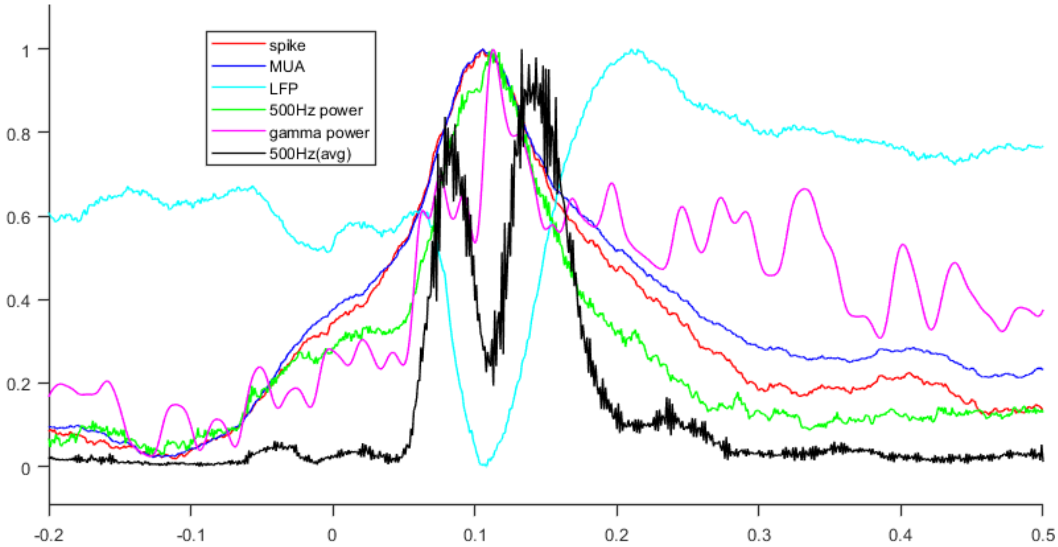

Figure S11: Time-resolved modulation of various neural signals obtained from microelectrode recordings of macaque V1. Microelectrode recordings from macaque V1 (averaged over 20 sessions, 4863 trials in total). Time-resolved activity is shown with respect to visual stimulus onset (time point 0). The panel shows the temporal evolution of the power (induced and evoked) in the 500 Hz band (+/- 50 Hz, assessed in periods of 50 ms shifted in steps of 1 ms), spiking activity (summed over 50 ms, in steps of 1 ms), the MUA (absolute Hilbert transformed bandpass filtered 750-8000 Hz data), the LFP (lowpass filtered at 500 Hz) and the gamma power (FFT, 60 Hz). The values are normalized for comparison.

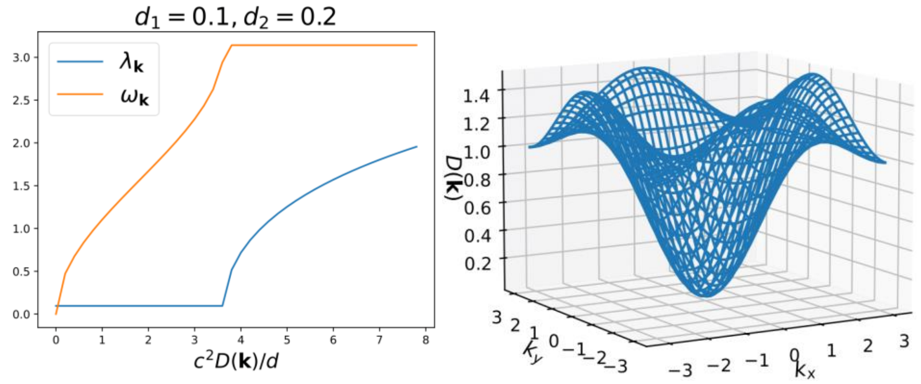

<u>Fig S12: Dependance of oscillation frequency and damping on the wavevector (wavelength).</u> Left: Dependence of the frequency ω_k and damping factor λ_k on the wavector k , given in terms of wavelength λ by $k = \frac{2\pi}{\lambda}$. The oscillation frequency increases with increasing wave-vector (decreasing wavelength) until it saturates to the value of π. The damping factor remains constant for low wave-vectors. After a certain threshold wave-vector k_{th}, the damping starts increasing with wave-vectors. Right: The dependence on the network topology enters through the factor $D(k)$, whose dependance on the wave-vector for the square grid with the next-nearest neighbour feedback is shown.

Table S1: Summary of model parameters and their values for simulations in this study.<br>This table provides the parameters and their respective values used in the simulations, enabling accurate reconstruction of the results. Note that parameter names may differ in the simulation scripts: c^2 = NI_slopev, f = slopeo_damping, and g = damping.
**Fig. 2A: input:** peak input 800 mV at 5 different locations; **coupling parameter**: NI_slopev 2.6, slopeo_damping 0.01, damping 0.0001; **model size:** 120x120
**Fig. 2B: input:** spontaneous activity 0.01% and 100 mV; **coupling parameter**: NI_slopev 2.6, slopeo_damping 0.01, damping 0.0001; **model size:** 3x3 - 151x151; **electrode diameter:** 2 mm; **signal length**: 3 s
**Fig. 3A: input:** periodic peak input of 7 Hz and 800 mV; **coupling parameter**: NI_slopev 2.6, slopeo_damping 0.01, damping 0.0001; **model size:** 120x120; **eletrode diameter**: 0.5 mm; **signal length**: 3s
**Fig. 3B: input:** sine input of 7 Hz and 800 mV; **coupling parameter**: NI_slopev 2.6, slopeo_damping 0.01, damping 0.0001; **model size:** 120x120; **electrode diameter**: 0.5 mm; **signal length**: 3s
**Fig. 3C: input:** 12 continuous periodic peak stimuli (29, 31 37, 41, 43, 47, 53, 59, 61, 67, 71, 73 Hz) and 100 mV; **coupling parameter**: NI_slopev 2, slopeo_damping 0.01, damping 0.0001; **model size:** 150x150; **electrode diameter**: 0.5 mm; **signal length**: 3s
**fig. S2: input:** 12 continuous periodic peak stimuli (29, 31 37, 41, 43, 47, 53, 59, 61, 67, 71, 73 Hz) and 100 mV; **coupling parameter:** NI_slopev 2.6655, slopeo_damping 0.01, damping 0.0001; **model size:** 150x150; **electrode diameter**: 0.5 mm; **signal length**: 3s
**Fig. 3D: input:** periodic peak input 7 Hz and 800 mV; **coupling parameter**: NI_slopev 0.01-3, slopeo_damping 0.01, damping 0.0001; **model size:** 150x150; **signal length**: 3s
**Fig. 3E: input:** periodic peak input 7 Hz and 800 mV; **coupling parameter**: NI_slopev 0.01-3, slopeo_damping 0.01, damping 0.0001; **model size:** 150x150; **signal length**: 3s
**Fig. 3F: input:** spontaneous activity 0.1% and 100 mV; **coupling parameter: (waking**: NI_slopev 2.6655, slopeo_damping 0.01; **SWS**: NI_slopev 0.1, slopeo_damping 0.01; **anesthesia**: NI_slopev 2.6655, slopeo_damping 0.01), damping 0.0001; **model size:** 150x150; **electrode diameter**: 0.5 mm; **signal length**: 3s
**Fig. 3G: input:** spontaneous activity 0.1% and 100 mV; **coupling parameter:** NI_slopev 2.6655, slopeo_damping 0.01, damping 0.0001; **model size:** 150x150; **electrode diameter**: 0.5 mm and 20 mm; **signal length:** 3s
**Fig. 3H: input:** spontaneous activity 0.01% and 100 mV; **coupling parameter:** NI_slopev 2.6655, slopeo_damping 0.01, damping 0.0001; **model size:** 150x150; **electrode diameter**: 20 mm; **signal length:** 9s; **stFT_windowsize**: 500ms
**fig. S3: input:** spontaneous activity 0.01% and 100 mV; **coupling parameter:** NI_slopev 0.1-2.6655, slopeo_damping 0.01, damping 0.0001; **model size:** 120x120; **electrode diameter**: 20 mm; **signal length:** 9s; **stFT_windowsize**: 500ms
**Fig. 3I: input:** spontaneous activity 0.01% and 100 mV; **coupling parameter:** NI_slopev 2.6655, slopeo_damping 0.01, damping 0.0001; **model size:** 150x150; **electrode diameter**: 0.5 mm; **electrode distances:** 0.5 (MEA1-2), 2 (MEA1-2), 4 (MEA1-3), 8 (MEA1-4), 16 (MEA1-5), 32 mm (MEA6); **signal length**: 3s
**Fig. 3J: input:** spontaneous activity 0.01% and 100 mV + sine burst of 200 ms at 100 Hz and 100 mV ; **coupling parameter:** NI_slopev 2.6655, slopeo_damping 0.01, damping 0.0001; **model size:** 150x150; **electrode diameter**: 0.5 mm; **electrode distances:** 0.5 (MEA1-2), 2 (MEA1-2), 4 (MEA1-3), 8 (MEA1-4), 16 (MEA1-5), 32 mm (MEA6); **signal length**: 3s

**Fig. 4: input:** spontaneous activity 0.1% and 100 mV and 21 bursts with random onset (every random(500) ms), random location, duration of random(100) ms, random HF (random(300-500) Hz) and random LF (random(30) Hz); **coupling parameter: (waking**: NI_slopev 2.6655, slopeo_damping 0.01; **SWS**: NI_slopev 0.1, slopeo_damping 0.01; **Ketamin anesthesia**: NI_slopev 2.6655, slopeo_damping 0.1; **Propofol anesthesia**: NI_slopev 0.1, slopeo_damping 0.1; **AD:** 2.6655, slopeo_damping 1% lesions**; schizophrenia:** 2.6655, slopeo_damping 0.01 + uncorrelated); **model size:** 150x150; **signal length**: 9s
**fig. S4: input:** spontaneous activity 0.1% and 100 mV and 21 bursts with random onset (every random(500) ms), random location, duration of random(100) ms, random HF (random(300-500) Hz) and random LF (random(30) Hz); **coupling parameter: (waking**: NI_slopev 2.6655, slopeo_damping 0.01; **SWS**: NI_slopev 0.1, slopeo_damping 0.01; **Ketamin anesthesia**: NI_slopev 2.6655, slopeo_damping 0.1; **Propofol anesthesia**: NI_slopev 0.1, slopeo_damping 0.1; **AD:** 2.6655, slopeo_damping 1% lesions**; schizophrenia:** 2.6655, slopeo_damping 0.01 + uncorrleated); **model size:** 150x150; **electrode diameter**: 0.5 mm; **signal length**: 9s
**Fig. S4: input:** spontaneous activity 1% and 100 mV; **coupling parameter**: NI_slopev 0.1-3.8, slopeo_damping 0.1, damping 0.0001; **model size:** 30x30 - 50x50; **electrode diameter**: 2 mm; **signal length**: 3 s
**fig. S6A: input:** spontaneous activity 0.1% and 100 mV and 21 bursts with random onset (every random(500) ms), random location, duration of random(100) ms, random HF (random(300-500) Hz) and random LF (random(30) Hz); **coupling parameter: (waking**: NI_slopev 2.6655, slopeo_damping 0.01; **SWS**: NI_slopev 0.1, slopeo_damping 0.01; **Ketamin anesthesia**: NI_slopev 2.6655, slopeo_damping 0.1; **Propofol anesthesia**: NI_slopev 0.1, slopeo_damping 0.1; **AD:** 2.6655, slopeo_damping 1% and 10% lesions**; schizophrenia:** 2.6655, slopeo_damping 0.01 + uncorrleated), damping 0.0001; **model size:** 150x150; **electrode diameter**: 20 mm; **signal length**: 9s
**Fig. S6B: input:** 12 continuous periodic peak stimuli (29, 31 37, 41, 43, 47, 53, 59, 61, 67, 71, 73 Hz) and 100 mV; **coupling parameter:** NI_slopev 2.6655, slopeo_damping 0.01, damping 0.0001; **model size:** 150x150; **electrode diameter**: 0.5 mm; **signal length:** 3s
**Fig. S6C: input:** 12 continuous periodic peak stimuli (29, 31 37, 41, 43, 47, 53, 59, 61, 67, 71, 73 Hz) and 100 mV; **coupling parameter:** NI_slopev 2.6655, slopeo_damping 0.01, damping 0.0001; **model size:** 150x150; **electrode diameter**: 0.5 mm; **signal length:** 3s
**Fig. S6D: input:** a sine wave burst of 50 ms containing a HF singal (>400 Hz, 400 mV) superimposed on a LF reference signal of 10 Hz (2400 mV) with onset every 1000 ms; **coupling parameter:** NI_slopev 2.6655, slopeo_damping 0.01, damping 0.0001, ratio_transmitted_amplitude 0.8, uncorrelated3_slopev 12.5%, uncorrelated4_slopeo 99%, uncorrelated1 99%; **model size:** 150x150; **electrode diameter**: 20 mm; **signal length:** 10s; **stFT_windowsize**: 500ms
**fig. S7: input:** spontaneous activity 0.01% and 100 mV; **coupling parameter:** NI_slopev 2.6655, slopeo_damping 0.01, damping 0.0001, ratio_neighbour_activation 0.8, ratio_inhibition_activation1 0.8-1; **model size:** 150x150; **electrode diameter**: 20 mm; signal length: 9s; **stFT_windowsize**: 500ms
**fig. S8: input:** spontaneous activity 0.01% and 100 mV; **coupling parameter: (A:** NI_slopev 2.6655, slopeo_damping 0.01, damping 0.0001, uncorrelated_ratio_nieghbour_activation 99%, ratio_transmitted_amplitude 1-0.5; **B**: NI_slopev 2.6655, slopeo_damping 0.01, damping 0.0001, uncorrelated3_slopev 12.5%, ratio_transmitted_amplitude 1-0.5; **C**: NI_slopev 2.6655, slopeo_damping 0.01, damping 0.0001, uncorrelated4_slopeo 99%, ratio_transmitted_amplitude 1-0.5); **model size:** 150x150; **electrode diameter**: 20 mm; signal length: 9s; **stFT_windowsize**: 500ms

**Fig. 5: input:** spontaneous activity 0.1% and 100 mV and 21 bursts with random onset (every random(500) ms), random location, duration of random(100) ms, random HF (random(300-500) Hz) and random LF (random(30) Hz); **coupling parameter: (waking**: NI_slopev 2.6655, slopeo_damping 0.001); **model size:** 150x150; **signal length**: as stated.
**fig. S10: input:** short burst chaos + grating stimulus of diameter 20 mm, 8 stripes, contrast 10 mV to -9 mV, onset every 3000 ms, stimulus length 1000 ms; **coupling parameter**: NI_slopev 2.6655, slopeo_damping 0.01, damping 0.0001; **model size:** 150x150; **electrode diameter**: 0.5 mm; **signal length**: 360s; **electrode distance to stimulus**: 30 mm; **stFT_windowsize**: 500ms
**Fig. 6: input:** short burst chaos + grating stimulus of diameter 20 mm, 8 stripes, contrast 10 mV to -9 mV, onset every 3000 ms, stimulus length 1000 ms; **coupling parameter**: NI_slopev 2.6655, slopeo_damping 0.01, damping 0.0001; **model size:** 150x150; **electrode diameter**: 0.5 mm; **signal length**: 360s; **electrode distance to stimulus**: 30, 40, 80 mm

Extended methods

Microelectrode recording in macaque monkey.

All procedures were approved by the ethics committee of the Radboud University, Nijmegen, NL.

Behavioral paradigm and visual stimulation.

A trained male macaque monkey participated in a study using a change detection paradigm. Trials started when the monkey touched a bar to start the experiment. A fixation point (~0.2° diameter) lit up and gaze had to be held within a small window around the fixation point, as previously described [2]. Consecutively, a pre-stimulus baseline (1 s) started, followed by the appearance of two stimuli placed in different visual quadrants (**fig. S8B**). We refer to this time point as stimulus onset. The stimuli were luminance gratings with a contrast of 100%, a diameter of 2–3°, a spatial frequency of 1–2 cycles/°, and a temporal frequency of 1–2°/s. The grating within the receptive field (RF) of the recorded neurons always had the preferred orientation and was moving. The grating outside the RF, on the opposite side of the fixation point, was either presented in the preferred or anti-preferred orientation and was also moving. The monkey had to detect a change of movement direction, which always happened on the side, which was not covered by the RF. Stimuli were presented on a 120 Hz CRT monitor. For the presented analysis, all trials were pooled independent of correct or incorrect responses and preferred or anti-preferred orientation outside the RF. The receptive fields were mapped in separate sessions, beforehand.

Surgery and recording.

Electrophysiological recordings were obtained from six to eight tungsten electrodes positioned in V1. Each electrode's signal was passed through a headstage (Plexon) amplified by a factor of 20. Signal acquisition, filtering, and amplification were done with a Neuralynx Digital Lynx acquisition system. This signal (sampling rate: 32,556 Hz) was used for further analysis.

ECoG recordings in macaque monkeys.

All procedures were approved by the ethics committee of the Radboud University, Nijmegen, NL.

Visual Stimulation and Attention Paradigm.

Two trained male macaque monkeys participated in a change detection task. Please note that these monkeys were different from the one participating in the V1 recording and that only data from one monkey (P) was analyzed. The experiment consisted of several conditions and a detailed description of the full experiment can be obtained from [3,4] as well as [5]). As for our

particular analysis, only one condition was used, the description will focus on this condition (**fig. S8B**). Additionally, please note that all trials were pooled independently of the behavioral outcome. Each trial started when the monkey touched a bar while a gray fixation point of 1 degree was shown at the center of the screen. This prestimulus baseline lasted 0.8 s. Then, two isoluminant and isoeccentric drifting sinusoidal gratings were presented, one in each visual hemifield (diameter: 3°, spatial frequency: ≈1 cycle/deg, drift velocity: ≈1 deg/s, resulting temporal frequency: ≈1 cycle/s, contrast: 100%). This is what we refer to as stimulus onset. In any given trial, one grating was tinted yellow, the other blue. These equiluminant colors were randomly assigned across trials. After 0.8–1.3 s, the fixation point changed color. This color served to indicate which grating was task-relevant. At random time points between stimulus onset and 4.5 s after cue onset, either the cued or the uncued grating was changed and the monkey should release the bar on detection of the change. The stimulus change consisted of a gentle bend of the stripes. Stimuli were presented on a 120 Hz CRT monitor (same as described above).

Surgery and recording.

Neuronal recordings were made from the left hemispheres through a micromachined 252-channel electrocorticogram-electrode (ECoG) array implanted subdurally [6]. The ECoG was placed directly onto the brain under anesthesia. For details on the procedure, please refer to [3]. Signals were obtained from the 252 electrode grid and were amplified 20 times by eight Plexon headstage amplifiers, then low-pass filtered at 8 kHz and digitized at 32 kHz by a Neuralynx Digital Lynx system.

Details of Mathematical Solution of the Network Model

Here we detail the mathematical solution of the network model. As mentioned in the main text, the network model can be described in terms of time evolution of the activation as a function of the node $A(x,t)$. The time evolution is given by the difference equation (a discrete analogue of a difference equation) given by eq. 1 in the main text (repeated for convenience):

$$D_t^2[A(x,t)] + (\delta_1 + \delta_2)D_t[A(x,t)] - c^2\nabla^2[A(x,t+1)] + \delta_1\delta_2\,A(x,t) = 0$$

equation S1

The equation is solved by first taking a spatial Fourier transform, to obtain $A(k,t)$, which gives us a decomposition of $A(x,t)$ into sinusoidal waves with wave-vector k. The wave-vector

k is related to the wavelength simply by $k = \frac{2\pi}{\lambda}$. Using the ansatz $A(k,t) = A(k)e^{i\,\omega t}$ for the time-evolution we get:

$$G^{-1}(k,\omega)A(k) = 0$$
$$G^{-1}(k,\omega) = \left(e^{i\,\omega} - e^{-\lambda_k}e^{i\omega_k}\right)\left(e^{i\,\omega} - e^{-\lambda_k}e^{-i\omega_k}\right)$$

equation S2

Postponing the question of values of ω_k and λ_k for a bit, we see that the general solution to the time evolution of the Fourier component $A(k,t)$ is given by

$$A^{\mathrm{gen}}(k,t) \;=\; e^{-\lambda_k t}(p_k\,\cos(\omega_k t) + q_k\,\sin(\omega_k t))$$

equation S3

The constants p_k and q_k ar generally determined by the state of the activationat early or late times (known as “boundary conditions”). We see that the component with wave-vector k oscillates with a frequency ω_k and are damped (exponentially suppressed in time) with a factor λ_k . Note that the Fourier modes $A^{\mathrm{gen}}\,(k,t)$ are the normal modes of this problem, i.e., they independently oscillate with normal frequencies $\omega_k + i\,\lambda_k$.

We now come to the explicit expressions for ω_k and λ_k, which specifies the frequency and damping factor as a function of the wave-vector and wavelength, consequently determining the speed of wave propagation. They are given by the imaginary and real parts of the expression:$\log\left(-\left(\frac{c^2}{d}D(k) + \delta_1 + \delta_2 - 2\right) \pm \left(-4(1 - \delta_1 - \delta_2 + \delta_1\delta_2) + \left(\frac{c^2}{d}D(k) + \delta_1 + \delta_2 - 2\right)^2\right)^{\frac{1}{2}}\right)$

equation S4 The dependence on the network topology (the graph or the lattice) appears only through the factor $D(k)$. For our case of the square lattice with next-nearest-neighbour feedback corresponds to

$$D(k) = \frac{1}{2}\left(\sin^2\left(\frac{k_x}{2}\right) + \sin^2\left(\frac{k_y}{2}\right) + \sin^2\left(\frac{k_x}{2} + \frac{k_y}{2}\right) + \sin^2\left(\frac{k_x}{2} - \frac{k_y}{2}\right)\right).$$

The behaviour of the frequency ω_k and the damping factor λ_k as a function of the wave-vector k is shown in fig. S12. For small k (large wavelengths), the damping is small and wavevector independent, while the oscillation frequency ω_k increases with increasing wave-vector. After

a threshold k_{th}, the damping factor λ_k starts increasing with increasing wave-vector (decreasing wavelengths), and the the waves start getting more strongly suppressed with time. At even higher wavevectors, the oscillation frequency saturates to π, which is the highest possible frequency on a discrete-time setting.

Now we come to the case of solving for the time-evolution given an additional stimulus $v(x,t)$. The evolution equation governing the network model is now given by

$$D_t^2[A(x,t)] + (\delta_1 + \delta_2)D_t[A(x,t)] - c^2\nabla^2[A(x,t+1)] + \delta_1\delta_2\, A(x,t)$$
$$= \quad \nabla^2[v(x,t+2)] + \frac{1}{1+g}\big(D_t[v(x,t+1)] + \delta_1 v(x,t+1)\big)$$

equation S5

To solve this, it is convenient to consider Fourier components $E(k,\omega)$ obtained from Fourier-transforming $E(x,t)$ along both space and time. The evolution equation becomes :

$$G^{-1}(k,\omega)A(k,\omega) = \overline{v}(k,\omega),$$

equation S6

where the additional source term $\overline{v}(k,\omega)$ is given in terms of the Fourier components $v(k,\omega)$ of the stimulus $v(x,t)$ by

$$\overline{v}(k,\omega) = v(k,\omega)\left(-\frac{c^2}{d}D(k)e^{i\omega} + \frac{1}{1+g}\left(e^{i2\omega} - e^{i\omega}\right) + \delta_2 e^{i\omega}\right)$$

equation S7

The full solution for Fourier components $A(k,\omega)$ can now be expressed as

$$A(k,\omega) = A^{\mathrm{gen}}(k,\omega) + A^{part}(k,\omega)\ .$$

equation S8

The general solution is the (Fourier transformed) damped oscillations described above, the ones we get without the stimulus, given in **eq. S3**. This gives us $G^{-1}(k,\omega)A^{\mathrm{gen}}(k,\omega) = 0$. $A^{\mathrm{part}}(k,\omega)$ generates the source term in **eq. S5**, i.e. , we want: $G^{-1}(k,\omega)A^{\mathrm{part}}(k,\omega) = \overline{v}(k,\omega)$.

This allows us to obtain

$$A^{\text{part}}(k,\omega) = G(k,\omega)\overline{v}(k,\omega)$$

equation S9

Combining **eq. S3**, **eq. S8** and **eq. S9**, we can obtain the full solution for $E(k,\omega)$ which can be inverse-Fourier-transformed to get the encoded signal $E(x,t)$. For the decoding problem , one tries to reconstruct the stimulus $v(x,t)$ from the output $E(x,t)$. In Fourier space, we have

$$A(k,\omega) = A^{\text{gen}}(k,\omega) + G(k,\omega)\overline{v}(k,\omega).$$

equation S10

Once the constants p_k and q_k in $E^{\text{gen}}(k,\omega)$ (**eq. S3**) are determined from the boundary conditions—for e.g. the condition that the activations were zero at early times before any stimulus was added, or would go to zero at very late times, $v(k,\omega)$ can be directly determined from this equation, and be inverse-Fourier transformed to get the real space stimulus $v(x,t)$. This completes our description of the encoding and decoding process. In general, to decode the input $v(x,t)$ on a grid of time points, one would require the output signal $E(x,t)$ on a comparably dense grid.

For the special case when the stimulus comprises of a static “image” function $I(x)$, which is added to the network for a fixed amount of time (either a single “kick”, or for an extended period). The stimulus is now known to decompose as $v(x,t) = I(x)f(t)$, where $f(t)$ is now a known function of time (perhaps describing a kick, or a step function describing the exposure time). The Fourier transform $v(k,\omega)$ then decomposes as $v(k,\omega) = I(k)f(\omega)$, with the function $f(\omega)$ known. Now, using eq. S7 and eq S10, knowing $E(k,t)$ at only one time point, but at all k (or equivalently at all nodes x) allows us to reconstruct $I(k)$ and , consequently via another inverse-Fourier transform, the static image $I(x)$.

Additional online information and data visualization

- PackageNetlogo.rar at https://www.biozentrum.uni-wuerzburg.de/bioinfo/computing/neuro and scripts at https://github.com/Department-of-Bioinformatics/non-local_cortex_simulation
  - code for our simulation (Netlogo Model)
  - tutorial for using the simulation
  - Matlab analysis scripts
  - example output
- supplementary **videos S1-S11**(https://www.biozentrum.uni-wuerzburg.de/bioinfo/computing/neuro) ,
  in particular we have:
  - simulation of anesthesia-like states (Ketamin, Propofol; **suppl. video S6** and **S7**)
  - disease modelling: Alzheimer's disease (AD; **suppl. video S8** and **S9**) and schizophrenia (**suppl. video S10**)

Supplementary references

1. Goonawardena, A.V., Morairty, S.R., Orellana, G.A., Willoughby, A.R., Wallace, T.L., and Kilduff, T.S. (2019). Electrophysiological characterization of sleep/wake, activity and the response to caffeine in adult cynomolgus macaques. Neurobiol Sleep Circadian Rhythms *6*, 9-23. 10.1016/j.nbscr.2018.08.001.
2. Womelsdorf, T., Fries, P., Mitra, P.P., and Desimone, R. (2006). Gamma-band synchronization in visual cortex predicts speed of change detection. Nature *439*, 733-736. 10.1038/nature04258.
3. Bosman, C.A., Schoffelen, J.M., Brunet, N., Oostenveld, R., Bastos, A.M., Womelsdorf, T., Rubehn, B., Stieglitz, T., De Weerd, P., and Fries, P. (2012). Attentional Stimulus Selection through Selective Synchronization between Monkey Visual Areas. Neuron *75*, 875-888. 10.1016/j.neuron.2012.06.037.
4. Bastos, A.M., Vezoli, J., Bosman, C.A., Schoffelen, J.M., Oostenveld, R., Dowdall, J.R., De Weerd, P., Kennedy, H., and Fries, P. (2015). Visual Areas Exert Feedforward and Feedback Influences through Distinct Frequency Channels. Neuron *85*, 390-401. 10.1016/j.neuron.2014.12.018.
5. Hindriks, R., Micheli, C., Bosman, C.A., Oostenveld, R., Lewis, C., Mantini, D., Fries, P., and Deco, G. (2018). Source-reconstruction of the sensorimotor network from resting-state macaque electrocorticography. Neuroimage *181*, 347-358. 10.1016/j.neuroimage.2018.06.010.
6. Rubehn, B., Bosman, C., Ostenveld, R., Fries, P., and Stieglitz, T. (2009). A MEMS-based flexible multichannel ECoG-electrode array. J Neural Eng *6*. 10.1088/1741-2560/6/3/036003.
